# N6-methyladenosine-mediated SH3BP5-AS1 upregulation promotes GEM chemoresistance in pancreatic cancer by activating the Wnt signaling pathway

**DOI:** 10.1186/s13062-022-00347-5

**Published:** 2022-11-17

**Authors:** Chengjie Lin, Yan Wang, Yihong Dong, Shihui Lai, Liang Wang, Shangeng Weng, Xiang Zhang

**Affiliations:** 1 Department of Hepatopancreatobiliary Surgery, The First Affiliated Hospital of Fujian Medical University, Fuzhou, 350001 Fujian China; 2 Fujian Abdominal Surgery Research Institute, The First Affiliated Hospital, Fujian Medical University, Fuzhou, 350001 Fujian China; 3 National Regional Medical Center, Binhai Campus of the First Affiliated Hospital, Fujian Medical University, Fuzhou, 350212 Fujian China

**Keywords:** SH3BP5-AS1, N6-methyladenosine, Pancreatic cancer, Chemoresistance, Wnt signaling pathway

## Abstract

**Background:**

Pancreatic cancer (PC) is highly malignant. Chemotherapy is the main treatment strategy, especially for patients with advanced PC. However, chemoresistance has always been a frequently encountered bottleneck. Hence, there is an urgent need to enhance the sensitivity of PC to gemcitabine (GEM).

**Results:**

We demonstrated that SH3BP5-AS1 was significantly upregulated in GEM-resistant PC and predicted a poorer prognosis. SH3BP5-AS1 stability was regulated by ALKBH5/IGF2BP1-mediated m6A modification. Loss of SH3BP5-AS1 reduced PC cell migration and invasion and enhanced the sensitivity of PC to GEM, as confirmed by gain- and loss-of-function assays in vitro and in vivo. Bioinformatics analysis revealed that SH3BP5-AS1 acted as a ceRNA against miR-139-5p and directly targeted CTBP1, affecting the biological behavior of PC cells. The mechanistic studies revealed that the upregulation of SH3BP5-AS1 increased CTBP1 expression by directly activating the Wnt signaling pathway, promoting GEM resistance.

**Conclusions:**

This study revealed that SH3BP5-AS1 activated Wnt signaling pathway by sponging miR-139-5p, upregulating CTBP1 expression, and contributing to the sensitivity of PC cells to GEM. SH3BP5-AS1 might be a potential target for PC therapy.

**Supplementary Information:**

The online version contains supplementary material available at 10.1186/s13062-022-00347-5.

## Background

Pancreatic cancer (PC) is highly malignant and is characterized by high recurrence rates and low resection rates. Its 5-year survival rate is only 9% [1]. The incidence rate of PC is increasing yearly. In 2018, the number of newly diagnosed cases of PC worldwide was similar to the number of deaths in the same year [2]. Currently, surgical resection combined with adjuvant chemotherapy is the standard treatment. Chemotherapy is the main treatment, especially for patients with advanced PC. Gemcitabine (GEM) is a first-line chemotherapy drug approved for the treatment of advanced PC, either alone or in combination with other chemotherapeutic agents [3]. Although in recent clinical trial combinations of GEM with other drugs resulted in relative improvements in median survival time, PC cells are less sensitive to GEM, resulting in unsatisfactory chemotherapy effects, owing to natural and/or acquired resistance to GEM [4]. Hence, there is an urgent need to enhance the sensitivity of PC to GEM. Therefore, it is important to explore the mechanisms underlying chemoresistance in PC and to optimize GEM formulations and medication regimens.

Long non-coding RNA (lncRNA) has been found to be involved in tumor chemoresistance in different pathways. LncRNAs PVT1, GSTM3TV2, and LINC00346 have been confirmed to promote GEM resistance in PC by competitively binding downstream genes [5–7]. All these studies indicated that lncRNAs were closely related to the chemoresistance of tumors. In this study, a patient-derived xenograft (PDX) model was used and genome sequencing was conducted. We found that the expression of SH3BP5-AS1 was positively correlated to the GEM resistance of PC, indicating an important role of SH3BP5-AS1 in the resistance of PC to GEM. Lina et al. found that the expression of SH3BP5-AS1 was upregulated in recurrent neuroblastoma and head and neck cancer [8, 9]. At present, few reports on SH3BP5-AS1 are available, and the role of LncRNA SH3BP5-AS1 in PC, including the causes for its abnormal expression in PC and the mechanism by which it affects the sensitivity to GEM, remains to be elucidated. In this study, We found that SH3BP5-AS1 could regulate CTBP1 in pancreatic cancer. CTBP1 was one member of the C-terminal binding proteins (CTBP) family which are identified to perform diverse functions in both developmental and oncogenic processes [10]. The abnormal function of CTBP were proved to be closely related to epithelial-mesenchymal transition (EMT), and cell apoptosis [11, 12]. However, CTBP has been little studied in pancreatic cancer, and the mechanism of its regulation is still unclear.

The N6-methyladenosine (m6A) modification has been confirmed to affect RNA stability, thereby affecting gene expression. In recent years, a large number of studies on m6A modifications of lncRNAs regulating tumor activity have been published. The m6A modification mainly occurs on the adenine residue in the RRACH sequence. Its functions are mainly characterized as “writer,” “eraser,” and “reader.” First, lncRNA is modified by a methylase “writer” (METTL3/14, WTAP, etc.) or a demethylase “eraser” (ALKBH5/FTO). Then it is recognized by a “reader” (YTHDF1/2/3, IGF2BP1, etc.) for stabilization or degradation [13]. In this study, we found that the expression level of lncRNA SH3BP5-AS1 is regulated by m6A modification, and its abnormal expression affects the sensitivity of PC to GEM. We further explored the potential effects of SH3BP5-AS1 on GEM resistance of PC and attempted to reduce GEM resistance of PC cells by regulating SH3BP5-AS1. We provide an experimental basis for the development of targeted drugs that enhance GEM sensitivity.

## Results

### SH3BP5-AS1 was upregulated in GEM-treated PDX pancreatic cancer

**Fig. 1:**
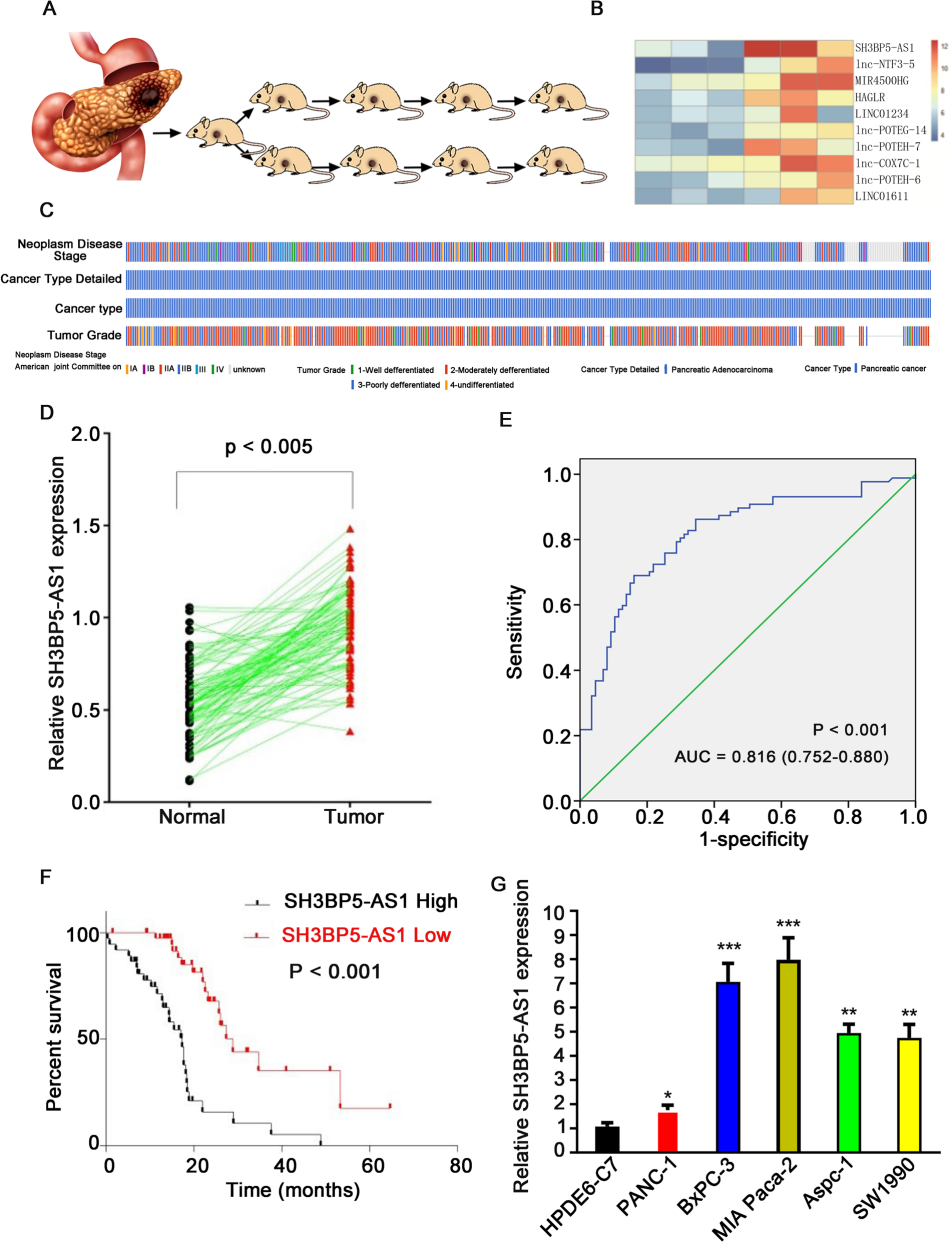
SH3BP5-AS1 was upregulated in GEM-treated PDX pancreatic cancer and its upregulation was negatively correlated with overall survival. **A** Establishment of the PDX pancreatic cancer model and experimental workflow of GEM treatment. **B** Heatmap displaying the top 10 upregulated lncRNAs in GEM-treated and control PDX mice. **C** Correlation analysis of SH3BP5-AS1 and clinicopathological features based on the TCGA database. **D** SH3BP5-AS1 was upregulated in pancreatic cancer tumor tissues compared with the paired adjacent tissues, as revealed by RT-qPCR. **E** SH3BP5-AS1 is an independent risk factor for the prognosis of pancreatic cancer, as revealed by ROC curve analysis. **F** Upregulation of SH3BP5-AS1 was correlated with poor overall survival. **G** SH3BP5-AS1 expression was significantly higher in pancreatic cancer cells compared with HPDE6-C7 cells. Data are shown as the mean ± SD. *P < 0.05; **P < 0.01

To explore the mechanism of GEM resistance in PC, we first established a PDX model, which was treated with either saline or GEM (Fig. 1A). We performed genomic sequencing of p4-pdx treated with GEM or the negative control to screen for hub genes. The top 10 differentially expressed lncRNAs are shown in Fig. 1B. The role of SH3BP5-AS1, one of the most strongly upregulated lncRNAs, in PC tumorigenesis and chemosensitivity was further investigated. The TCGA database suggested that SH3BP5-AS1 is closely related to the tumor grade of PC (Fig. 1C). Analysis of the correlation between SH3BP5 expression and clinicopathological data revealed that high SH3BP5-AS1 expression was closely related to tumor TNM stage, tumor size, and degree of differentiation in our center (Table 1). SH3BP5-AS1 expression was measured in 87 pair-matched PC tumor and adjacent normal tissues by RT-qPCR, and the results suggested that SH3BP5-AS1 was significantly upregulated in tumor tissue compared to normal tissue (Fig. 1D). ROC curve analysis revealed that it was an independent risk factor for the prognosis of PC (Fig. 1E). The survival of patients with low SH3BP5-AS1 expression was significantly better than that of patients with high SH3BP5-AS1 expression (Fig. 1F). At the same time, the expression levels of SH3BP5-AS1 in five PC cell lines and normal pancreatic cells were detected in vitro. SH3BP5-AS1 expression was significantly higher in PC cells than in normal pancreatic cells (Fig. 1G). Collectively, the above results demonstrated that SH3BP5-AS1 plays a key role in PC tumorigenicity and might serve as a key regulator of GEM resistance.

**Table 1:** Relationship between SH3BP5-AS1 expression and clinicopathological parameters in 87 PC patients

Variables	All cases	SH3BP5-AS1 expression		*P*
		Low (*n* = 31)	High (*n* = 56)	
*Age (years)*				
< 50	40	14	26	0.910
≥ 50	47	17	30	
*Gender*				
Male	59	22	37	0.640
Female	28	9	19	
*Alcohol history*				
No	41	14	27	0.785
Yes	46	17	29	
*Smoking*				
No	37	12	25	0.592
Yes	50	19	31	
*CEA*				
Normal	17	9	8	0.097
High	70	22	48	
*CA199*				
Normal	26	12	14	0.181
High	61	19	42	
*Tumor size (cm)*				
< 4	52	23	29	0.041^*^
≥ 4	35	8	27	
*TNM stage*				
I-II	31	16	15	0.02^*^
III-IV	56	15	41	
*Lymph node invasion*				
Absent	61	26	35	0.035^*^
Present	26	5	21	
*Metastasis*				
No	40	19	21	0.034^*^
Yes	47	12	35	

^*^P < 0.05

### Abnormal expression of SH3BP5-AS1 was involved in the invasion, migration, and stemness of pancreatic cancer cells

**Fig. 2:**
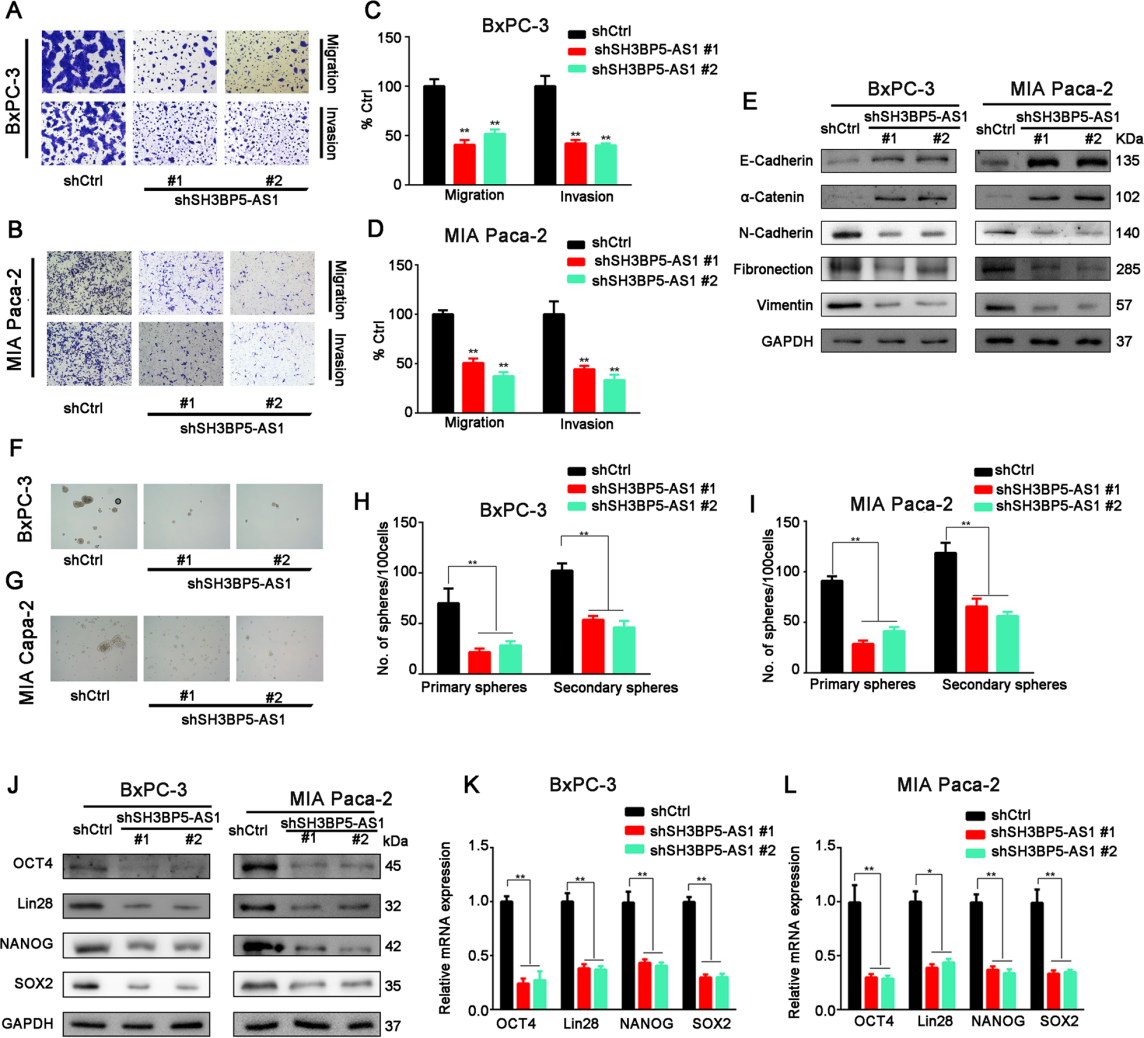
Silencing of SH3BP5-AS1 expression inhibited the invasion, migration, and stemness of pancreatic cancer cells in vitro. vivo. **A**, **B** Transwell assay revealed the migration and invasion abilities of BxPC-3 and MIA Paca-2 cells transfected with shCtrl or shSH3BP5-AS1. **C**, **D** Histogram displaying the statistical results of the Transwell assay. **E** EMT-related markers were detected by western blot. **F**–**I** Sphere formation analysis. **J**–**L** Stem cell-like cell markers Lin28, OCT4, NANOG, and SOX-2 were detected by western blot and RT-qPCR. Data are shown as the mean ± SD of three replicates. *P < 0.05; **P < 0.01

To further explore the effects of SH3BP5-AS1 on the biological behavior of PC cells, PC cells (BxPC3, MIA Paca-2, and PANC-1) were transfected with lentiviral vectors encoding SH3BP5-AS1 inserts or short hairpin RNA (shRNA) and Transwell and sphere formation assays were conducted. The transfection efficiency of shSH3BP5-AS1#1, shSH3BP5-AS1#2 and SH3BP5-AS1 overexpression plasmid was shown in Additional file 1: Fig. S1. After silencing SH3BP5-AS1, the invasion, migration, and sphere formation abilities of PC cells (BxPC3, MIA Paca-2) were significantly reduced (Fig. 2A–D and F–I). On the contrary, overexpression of SH3BP5-AS1 enhanced the above cellular behaviors (Additional file 1: Fig. S2A–E).

Epithelial-to-mesenchymal transition (EMT) has been proved to be closely related to cell invasion and migration [14]. Therefore, we also detected EMT-related proteins. The results showed that the epithelial markers E-cadherin and α-catenin were upregulated, whereas the levels of the mesenchymal markers N-cadherin, fibronectin, and vimentin were decreased when SH3BP5-AS1 was silenced (Fig. 2E). The opposite results were obtained when SH3BP5-AS1 was overexpressed in PC cells (Additional file 1: Fig. S2C). Stem cell-like cell markers Lin28, OCT4, NANOG, and SOX-2 have been found to be related to cell stemness [15], and these proteins and mRNA were significantly downregulated when SH3BP5-AS1 was silenced and upregulated when SH3BP5-AS1 was overexpressed (Fig. 2J-L, Additional file 1: Fig. S2F and G). These findings suggested that SH3BP5-AS1 stimulates PC cell migration, invasion, and stemness.

### SH3BP5-AS1 promotes pancreatic cancer cell resistance to gemcitabine in vitro and in vivo

**Fig. 3:**
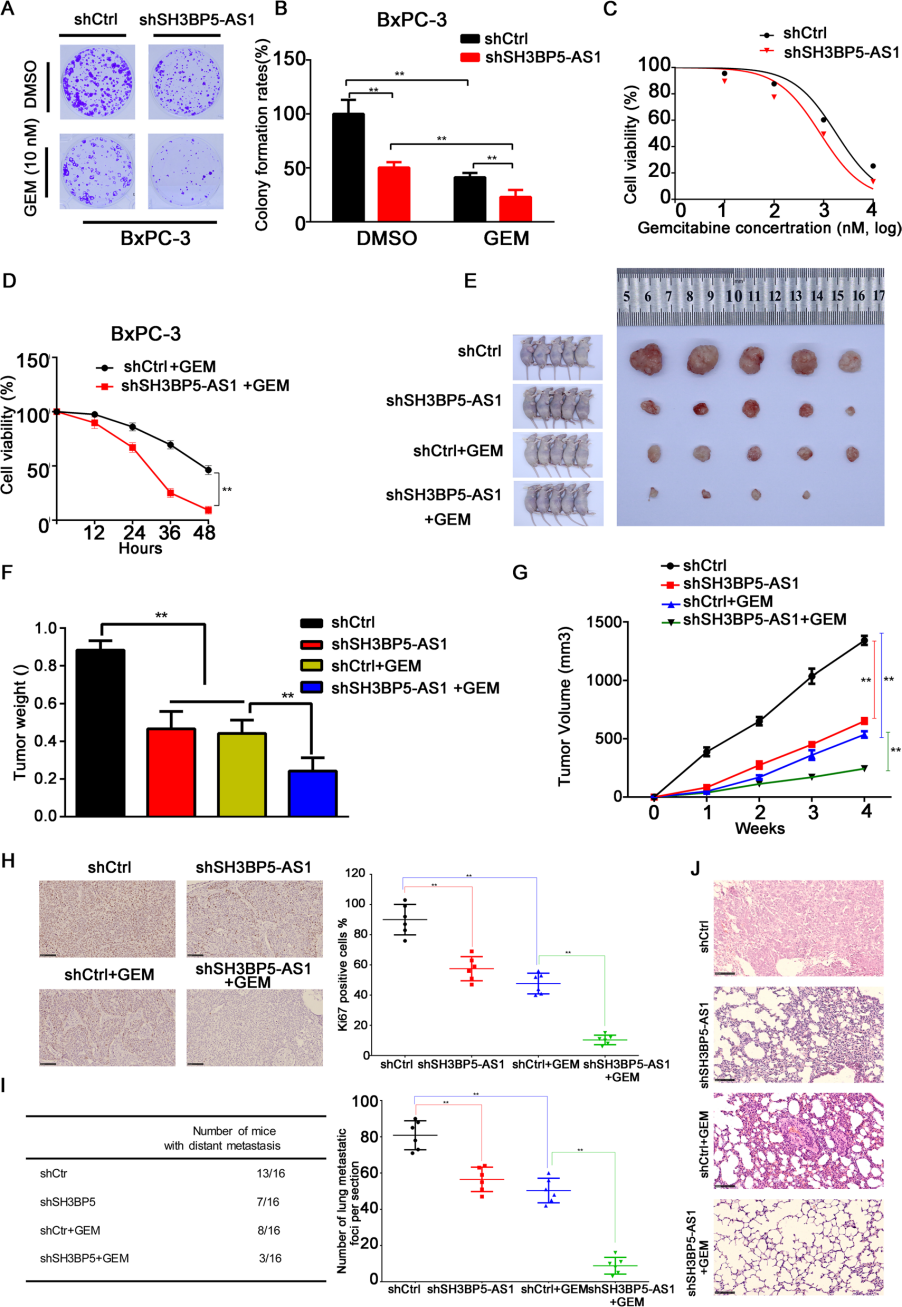
Upregulation of SH3BP5-AS1 promotes pancreatic cancer cell resistance to gemcitabine in vitro and in vivo. **A**, **B** Colony formation in BxPC-3 cells transfected with shCtrl or shSH3BP5-AS1 and treated with DMSO or GEM. **C** BxPC-3 cells with stable silencing of SH3BP5-AS1 were treated with GEM at different concentrations for 48 h. Cell viability was measured by MTT assay. **D** Stable SH3BP5-AS1-silenced BxPC-3 cells were treated with GEM (1 μM) for 48 h. Cell viability was measured by MTT assay at different timepoints. **E** Representative images of the xenograft tumors of BxPC-3 cells with stable SH3BP5-AS1 silencing in nude mice following GEM or saline treatment. **F**, **G** Histograms displaying the tumor weight and volume. **H** Ki67 expression in BxPC-3 cells with stable SH3BP5-AS1 silencing in nude mice after GEM or saline treatment. **I** Table summarizing the results of lung metastasis. **J** Representative images of H&E staining of the xenograft tumors. Data are shown as the mean ± SD. *P < 0.05; **P < 0.01

These above results strongly supported the notion that SH3BP5-AS1 is involved in PC carcinogenicity; however, whether SH3BP5-AS1 affects GEM chemoresistance in PC still remained to be elucidated. First, lentiviral vectors encoding SH3BP5-AS1 inserts or shRNA were transfected into BxPC3 and MIA Paca-2 cells. Then the cells were treated with GEM or DMSO for 72 h. We found that the PC cell proliferation ability was significantly reduced when SH3BP5-AS1 was silenced compared to the negative control group (Fig. 3A and B, Additional file 1: Fig. S3A and B). Moreover, SH3BP5-AS1 silencing enhanced the inhibitory effect of GEM on PC cells (Additional file 1: Fig. S3E and F). To corroborate the above results, cell viability was tested after exposing the cells to different GEM concentrations for 48 h. The results showed that silencing of SH3BP5-AS1 enhanced the sensitivity of PC cells to GEM. Compared with the control group, the IC_50_ value of GEM was significantly decreased in the SH3BP5-AS1 silencing group (Fig. 3C, Additional file 1: Fig. S3C). On the contrary, after overexpression of SH3BP5-AS1, the sensitivity of the PC cell line PANC-1 to GEM was significantly reduced (Additional file 1: Fig. S3E–H). Furthermore, we found that silencing of SH3BP5-AS1 reduced the viability of GEM-treated PC cells in a time-dependent manner (Fig. 3D, Additional file 1: Fig. S3D).

We further explored the effects of SH3BP5-AS1 on the sensitivity of PC to GEM through in vivo experiments. Stably silenced SH3BP5-AS1 PC cell lines and control cell lines were injected subcutaneously and via the tail vein, and the mice received 50 mg/kg GEM or saline. After 4 weeks, a significantly lower tumor volume and weight were observed in the SH3BP5-AS1 silencing group, after treatment with either GEM or saline, especially in the GEM treatment group (Fig. 3E–G). The Ki67 immunostaining results suggested that silencing of SH3BP5-AS1 inhibits proliferation and enhances GEM sensitivity of PC cells (Fig. 3H). In the lung metastasis model, we found that silencing of SH3BP5-AS1 significantly reduced the distant metastasis of the tumor (7/16) compared with the control group (13/16). Interestingly, compared with the GEM treatment group, silencing of SH3BP5-AS1 can significantly improve the efficacy of GEM, which reduced the lung metastasis rate and the number of lung metastases (Fig. 3I and J). The above results suggested that SH3BP5-AS1 silencing enhanced the sensitivity of PC cells to GEM both in vitro and in vivo.

### ALKBH5/IGF2BP1-mediated m6A modification contributed to SH3BP5-AS1 overexpression

**Fig. 4:**
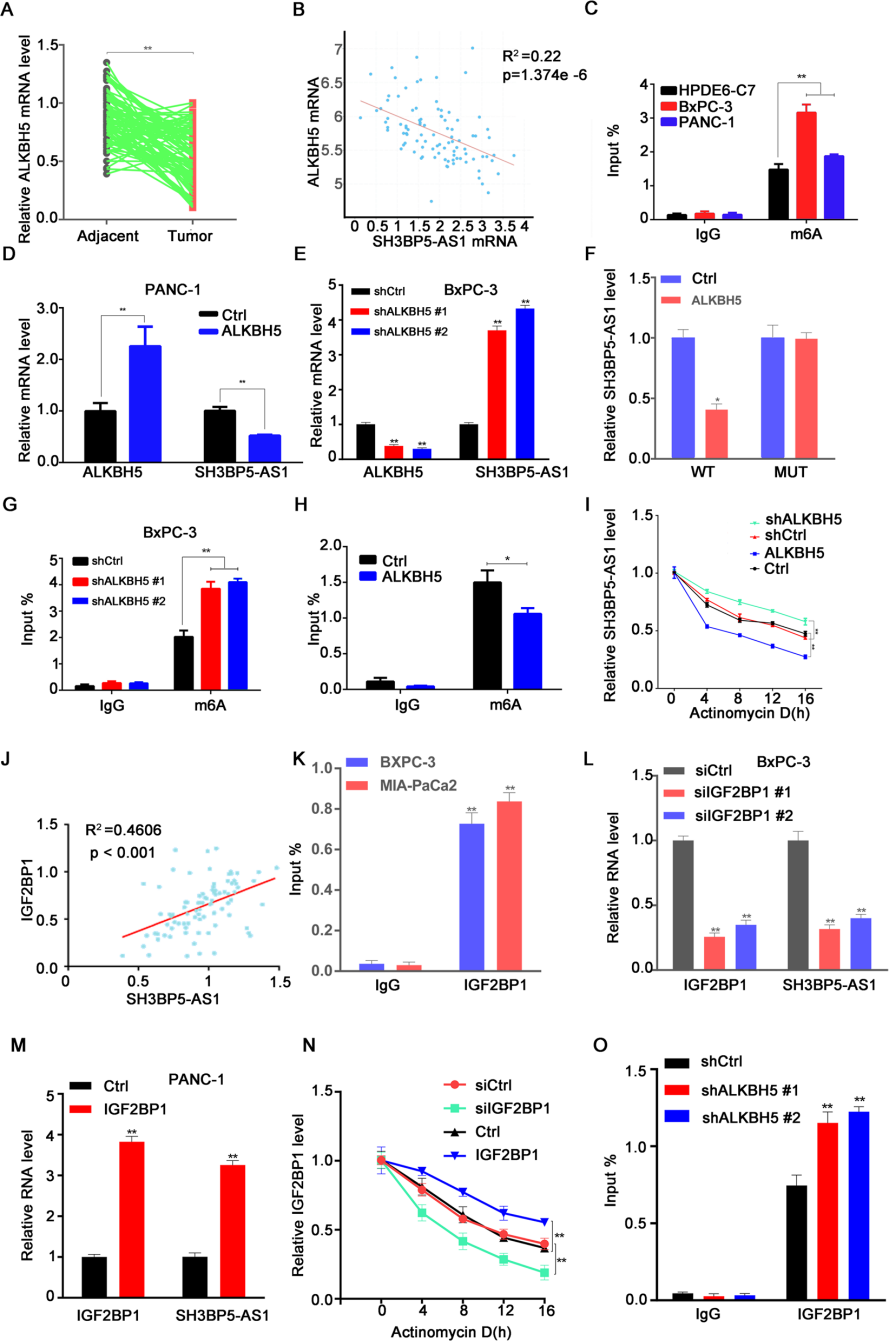
ALKBH5/IGF2BP1-mediated m6A modification contributed to SH3BP5-AS1 overexpression. **A** ALKBH5 was downregulated in pancreatic cancer tumor tissues compared with the paired adjacent tissues, as revealed by RT-qPCR. **B** The negative correlation between the levels of ALKBH5 and SH3BP5-AS1 was confirmed using the TCGA database. **C** The RIP RT-qPCR assay revealed that m6A modification of SH3BP5-AS1 was mainly enriched in pancreatic cancer cells (BxPC-3, PANC-1) compared with HPDE6-C7. **D**, **E** The expression level of SH3BP5-AS1when ALKBH5 was overexpressed or silenced. **F** The relationship between ALKBH5 and SH3BP5-AS1 was confirmed by the luciferase reporter assay. **G**, **H** RIP RT-qPCR results showing the m6A modification level of SH3BP5-AS1 when ALKBH5 was overexpressed or silenced. **I** SH3BP5-AS1 stability analysis in BxPC-3 cells with ALKBH5 overexpression or knockdown in the presence of actinomycin D. **J** The negative correlation between the levels of IGF2BP1 and SH3BP5-AS1 in pancreatic cancer cells. **K** The enrichment of IGF2BP1 on SH3BP5-AS1 in pancreatic cancer cells was analyzed by RIP RT-qPCR assay. **L**, **M** The expression level of SH3BP5-AS1 when IGF2BP1 was overexpressed or knocked down. **N** Actinomycin D assays showing the effects of ALKBH5 overexpression or knockdown on IGF2BP1 stability. **O** RIP RT-qPCR assay results showing the enrichment of IGF2BP1 on SH3BP5-AS1 upon ALKBH5 silencing. Data are shown as the mean ± SD. *P < 0.05; **P < 0.01

m6A modification has been confirmed to be involved in the regulation of RNA expression [16]. m6A modifications are placed and removed by methylases and demethylases, respectively. ALKBH5, a demethylase, is well known to be involved in the occurrence and development of various cancers [17–19]. In this study, we found that ALKBH5 was significantly downregulated in tumor tissues compared to the adjacent tissues (Fig. 4A), and its expression was negatively correlated with SH3BP5-AS1 levels in tumor tissues (Fig. 4B). m6A RIP results showed that m6A levels in PC cell lines (BxPC-3 and PANC-1) were significantly higher than in normal pancreatic cells (HPDE6-C7) (Fig. 4C). The in vitro results also revealed that SH3BP5-AS1 was significantly downregulated while ALKBH5 was overexpressed; on the contrary, SH3BP5-AS1 upregulation was observed after ALKBH5 was silenced (Fig. 4D and E). The dual luciferase report assay indicated that ALKBH5 could attenuate luciferase activity in the wild-type SH3BP5-AS1 group; however, luciferase activity was not affected in the mutant SH3BP5-AS1 group (Fig. 4F). MeRIP assays showed that ALKBH5 silencing increased the m6A modification level of SH3BP5-AS1 (Fig. 4G), whereas overexpression of ALKBH5 decreased the m6A modification level of SH3BP5-AS1 in PC cells (Fig. 4H). The actinomycin D assay (a drug that blocks de novo RNA synthesis) showed that ALKBH5 promoted SH3BPH5-AS1 degradation, while silencing of ALKBH5 contributed to the stability of SH3BP5-AS1 (Fig. 4I). Collectively, ALKBH5 acted as an important “eraser” in regulating SH3BP5-AS1. However, an “eraser” cannot regulate SH3BP5-AS1 expression alone; it also requires a “reader.” Studies have found that ALKBH5-mediated m6A demethylation inhibited mRNA transcription by the m6A effector IGF2BP1 [18, 20]. Our correlation analysis revealed that IGF2BP1 expression was positively correlated with SH3BP5-AS1 levels (Fig. 4J). We hypothesized that IGF2BP1 acted as a “reader” for the regulation of SH3BP5-AS1. To confirm the above hypothesis, the RIP assay was performed to affirm the direct binding between IGF2BP1 and SH3BP5-AS1 (Fig. 4K). SH3BP5-AS1 expression was significantly decreased after IGF2BP1 silencing and increased after IGF2BP1 overexpression (Fig. 4L and M). The results of our actinomycin D assay confirmed the above conclusion (Fig. 4N). Then an RIP assay was conducted to explore the correlation between the “eraser” ALKBH5 and the “reader” IGF2BP1. The results suggested that IGF2BP1 expression was negatively correlated with ALKBH5 levels (Fig. 4O). In conclusion, low expression of the “eraser” ALKBH5 maintains a high m6A modification level of SH3BP5-AS1, which further promotes the recognition of m6A-SH3BP5-AS1 by the “reader” IGF2BP1.

### miR-139-5p is involved in the oncogenic roles of SH3BP5-AS1 in PC

**Fig. 5:**
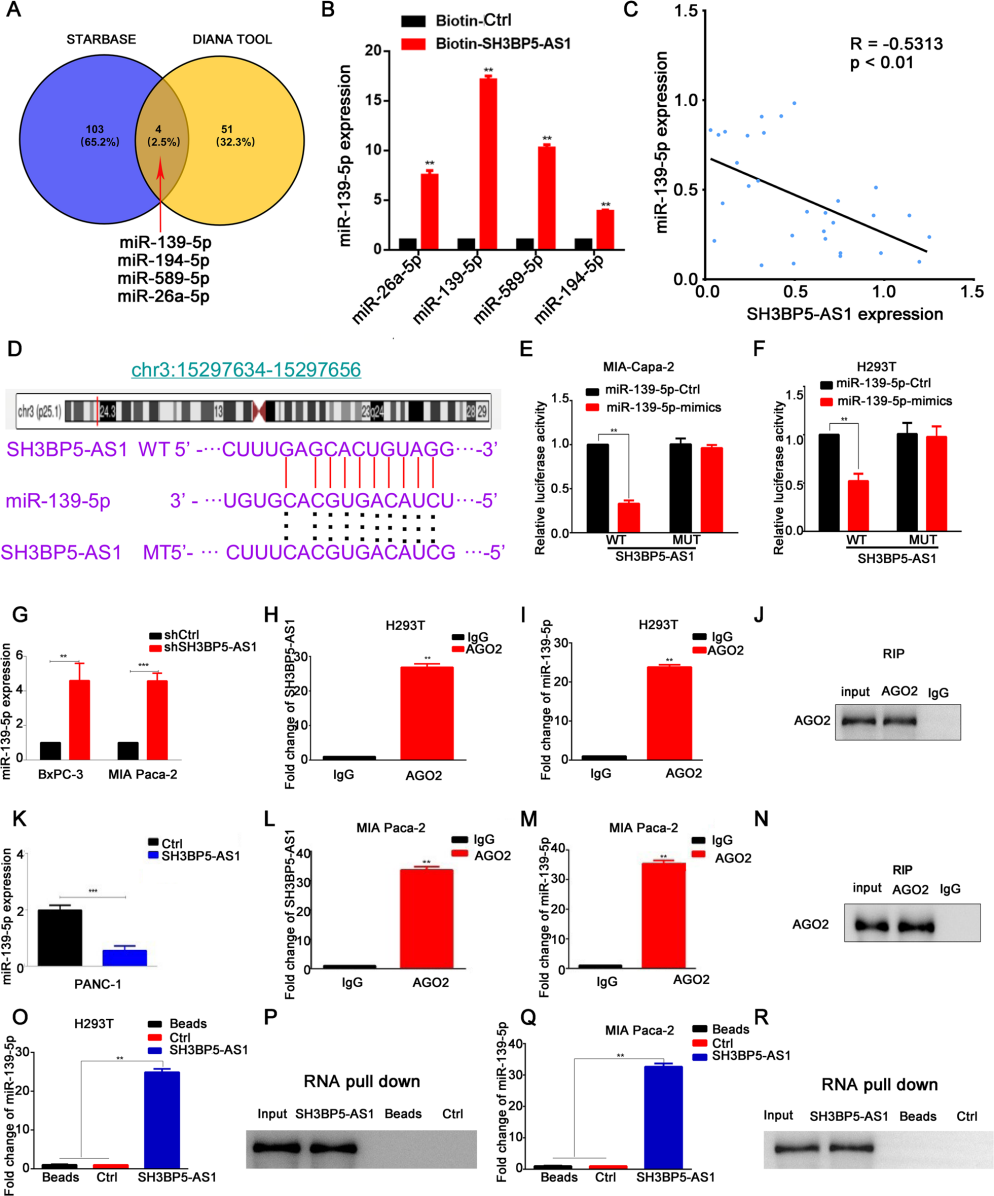
miR-139-5p is involved in the oncogenic roles of SH3BP5-AS1 in pancreatic cancer. **A** Venn diagram showing the potential target miRNAs of SH3BP5-AS1 based on the public database ENCORI and the DIANA Tool. **B** RNA pulldown assay was performed to prove miRNA-139-5p was the most enriched in the precipitate of SH3BP5-AS1 than other miRNA candidates. **C** Correlation analysis of SH3BP5-AS1 and miRNA-139-5p. **D** Schematic diagram of the binding sites between SH3BP5-AS1 and miRNA-139-5p. **E**, **F** A dual luciferase reporter assay was conducted to determine the relative luciferase activities in pancreatic cancer cells following transfection with the indicated constructs. **G** miRNA-139-5p was overexpressed following silencing of SH3BP5-AS1 in BxPC-3 and MIA Paca-2 cells. **H**–**J** An RIP assay was conducted to evaluate the relative enrichment of SH3BP5-AS1 and miRNA-139-5p in anti-IgG- or anti-AGO2-specific immunoprecipitates in H293T cells. **K** miRNA-139-5p was downregulated following SH3BP5-AS1 overexpression in PANC-1 cells. **L**–**N** An RIP assay was conducted to evaluate the relative enrichment of SH3BP5-AS1 and miRNA-139-5p in anti-IgG- or anti-AGO2-specific immunoprecipitates in MIA Paca-2 cells. **O**–**R** An RNA pull-down assay was conducted to detect the interaction among LNCAROD, miR-145-5p, and AGO2 in H293T and MIA Paca-2 cells. Data are shown as the mean ± SD. *P < 0.05; **P < 0.01

It has been reported that lncRNAs exert carcinogenic effects through acting as ceRNAs [21–23]. Here, we explored whether this is also the case for SH3BP5-AS1. First, Combined with the public database ENCORI, previously known as starBase v2.0, and the DIANA Tool, miRNA-194-5p, miRNA-589-5p, miRNA-26a-5p and miRNA-139-5p were predicted to be a target miRNA of SH3BP5-AS1 (Fig. 5A). Furthermore, RNA pulldown assay was performed to prove miRNA-139-5p was the most enriched in the precipitate of SH3BP5-AS1 than other miRNA candidates (Fig. 5B). Therefore, miRNA-139-5p was selected for follow-up study. RT-qPCR was performed to further prove the interaction between miRNA-139-5p and SH3BP5-AS1. The results suggested that SH3BP5-AS1 expression is negatively correlated with miRNA-139-5p levels (Fig. 5C). The results of our luciferase assays confirmed that in both H293T and PC cells, miR139-5p significantly decreased the luciferase activity of wild-type SH3BP5-AS1 but did not affect that of mutant SH3BP5-AS1 (Fig. 5D–F). Furthermore, silencing of SH3BP5-AS1 can significantly upregulate the expression of miR139-5p, whereas the opposite results were obtained after SH3BP5-AS1 overexpression (Fig. 5G and K). The above results indicated that SH3BP5-AS1 might sponge miR139-5p, thereby affecting downstream target genes. The relationship was verified by RIP assay. We found that both SH3BP5-AS1 and miR139-5p were enriched in AGO2 precipitate (Fig. 5H–J, L–N). The results of our RNA pull-down assays confirmed the above results. miR-139-5p and AGO2 were more strongly enriched in the biotin-labeled SH3BP5-AS1 group than in the control group (Fig. 5O–R). These results indicated that SH3BP5-AS1/miR-139-5P participate in the carcinogenic mechanism of PC.

To further explore the effect of SH3BP5-AS1/miRNA-139-5p on the biological behavior of PC and GEM resistance, first of all, Transwell and sphere formation assays proved that silencing of SH3BP5-AS1 could reduce the invasion, migration, and spheroidizing abilities of PC cells, while miRNA-139-5p inhibition could reverse the effect of shSH3BP5-AS1 on PC cells. On the contrary, overexpression of miRNA-139-5p could reverse the carcinogenic effects of SH3BP5-AS1 (Additional file 1: Fig. S4A–I). In the drug resistance intervention experiment, we found that miRNA-139-5p inhibition could reverse the effect of shSH3BP5-AS1 on GEM sensitivity of PC cells. Overexpression of miRNA-139-5p increased the sensitivity of PC cells to GEM. This further illustrates that SH3BP5-AS1 can affect the biological behavior and GEM resistance of PC cells through miRNA-139-5p (Additional file 1: Fig. S4J–O).

### miR-139-5p sponging activity decreases CTBP1 levels, affecting the biological behavior of PC cells

**Fig. 6:**
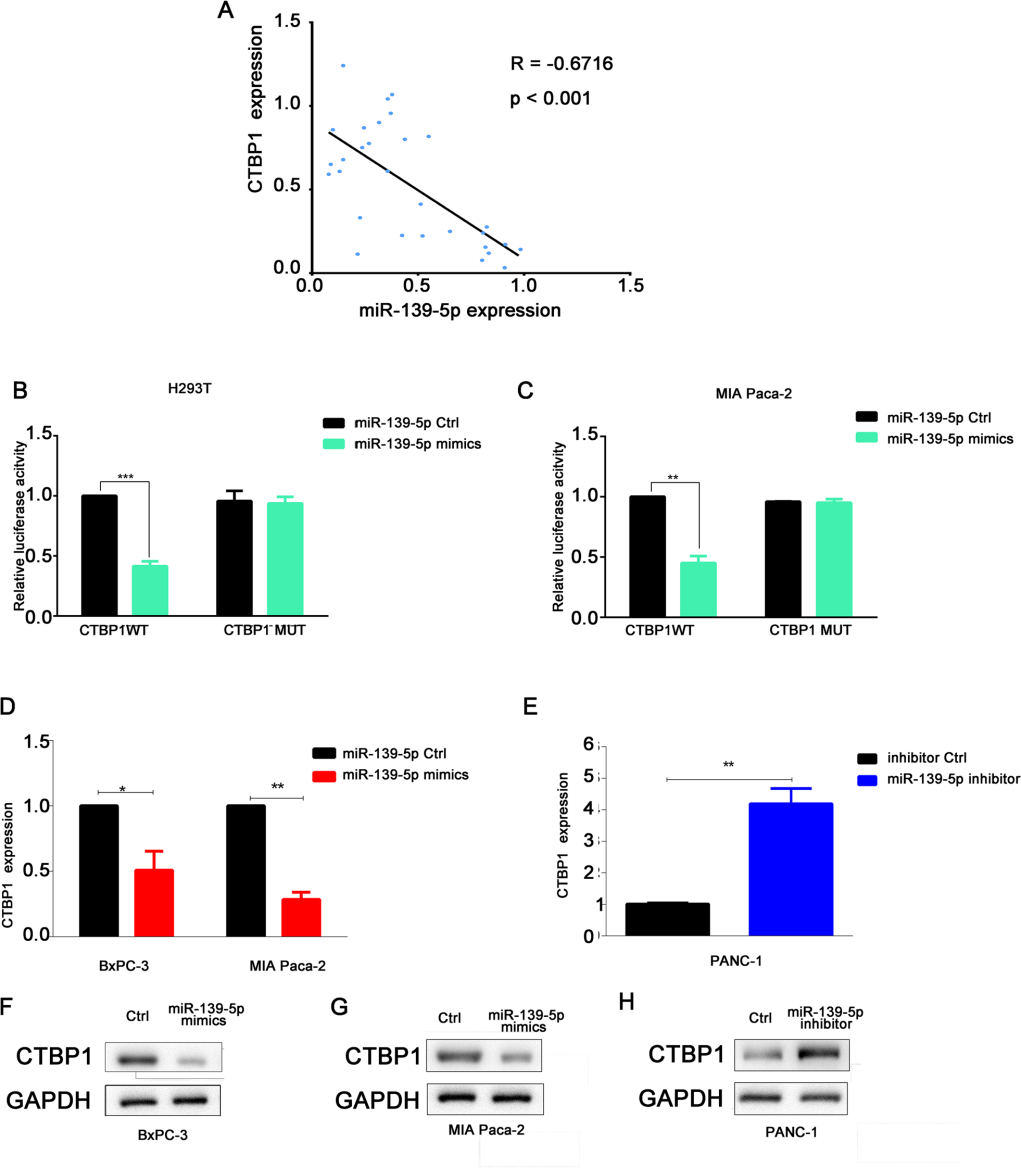
miR-139-5p negatively regulated the expression of the target gene *CTBP1*. **A** Correlation analysis of CTBP1 and miRNA-139-5p. **B**, **C** A dual luciferase reporter assay was conducted to determine the relative luciferase activities following the indicated transfections in H293T and MIA Paca-2 cells. **D**, **E** CTBP1 was downregulated following miRNA-139-5p overexpression in BxPC-3 and MIA Paca-2 cells and overexpressed following silencing of miRNA-139-5p in PANC-1 cells. **F**–**H** CTBP1 protein expression was detected following the indicated transfections in BxPC-3, MIA Paca-2, and PANC-1 cells. Data are shown as the mean ± SD. *P < 0.05; **P < 0.01

**Fig. 7:**
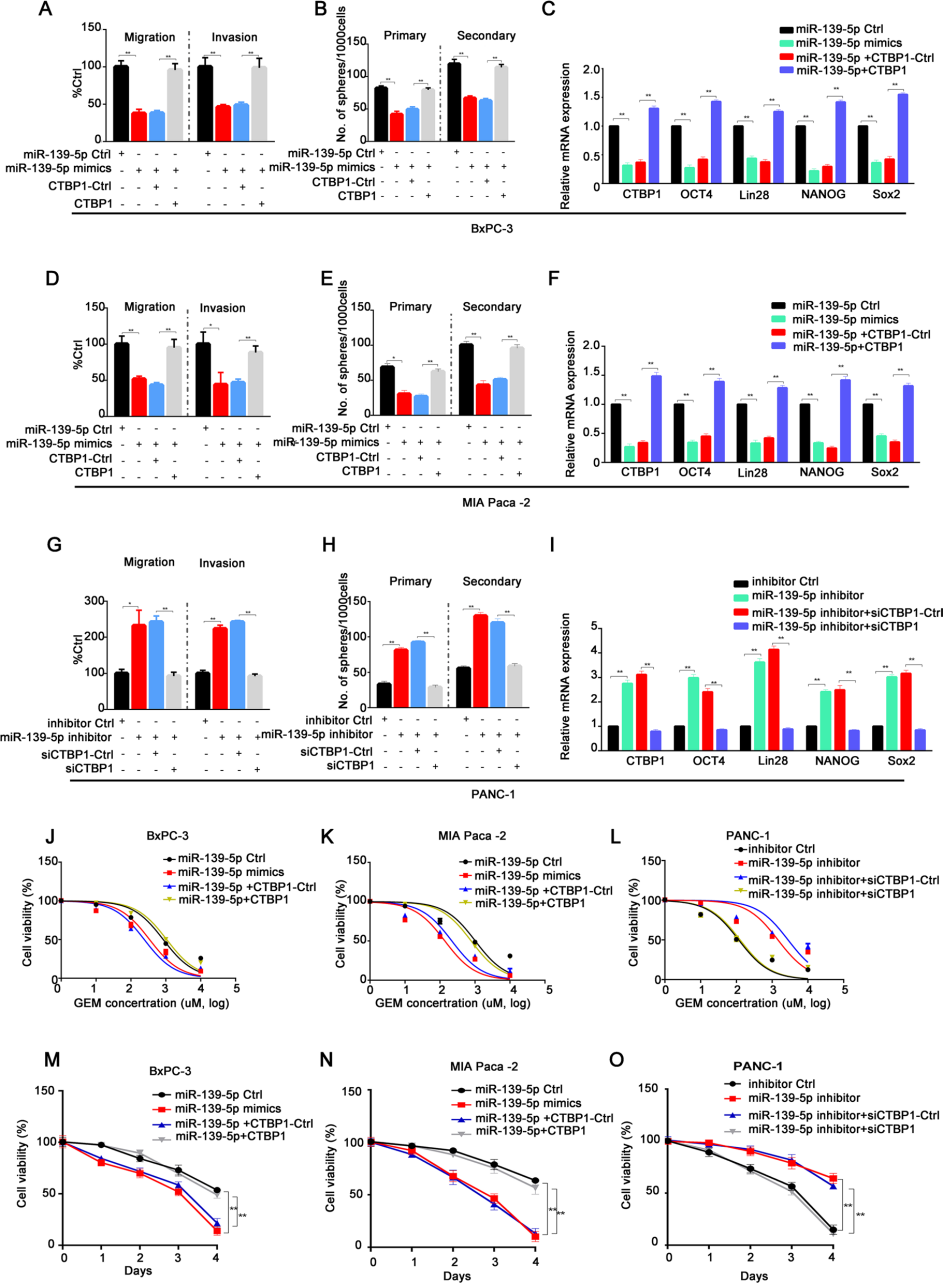
miR-139-5p sponging activity decreases CTBP1 levels, affecting the biological behavior of pancreatic cancer. **A**, **B** Transwell and Sphere formation assays were performed in BxPC-3 cells after co-transfection with miRNA-139-5p mimics and/or CTBP1 overexpression plasmid. **C** Stem cell-like cell markers Lin28, OCT4, NANOG, and SOX-2 were detected by western blot following the indicated transfections in BxPC-3 cells. **D**–**E** Transwell and Sphere formation assays were performed in MIA Paca-2 cells following the indicated transfections. **F** Stem cell-like cell markers Lin28, OCT4, NANOG, and SOX-2 were detected by western blot following the indicated transfections in MIA Paca-2 cells. **G**, **H** Transwell and Sphere formation assays were performed in PANC-1 cells after co-transfection with miRNA-139-5p-shRNA and CTBP1-shRNA. **I** Stem cell-like cell markers Lin28, OCT4, NANOG, and SOX-2 were detected by western blot following the indicated transfections in PANC-1 cells. **J**–**L** Pancreatic cancer cells with indicated transfections were treated with GEM at different concentrations for 48 h. Cell viability was measured by MTT assay. **M**–**O** Pancreatic cancer cells with indicated transfections were treated with GEM (1 μM) for 48 h. Cell viability was measured by MTT assay at different timepoints. Data are shown as the mean ± SD. *P < 0.05; **P < 0.01

As shown above, miR-139-5p could inhibit the expression of downstream target genes by forming a RISC complex with AGO2. Transcriptome sequencing was used to further screen downstream target genes. As shown in Additional file 1: Fig. S5A and B, we found that CTBP1 was significantly downregulated when miR-139-5P was overexpressed. We conducted functional enrichment and signal pathway analyses of differentially expressed genes and found that genes, includi ng *CTBP1*, are mainly enriched in Wnt signaling (Additional file 1: Fig. S5C and D). Based on the miR-TARGET database to predict miRNA target genes, it was found that there were a total of 218 genes that could be regulated by miR-139-5p. Among the overlapping genes, the difference in *CTBP1* expression is particularly obvious (Additional file 1: Fig. S5E), indicating that *CTBP1* might be a hub gene downstream of miR-139-5p. To prove this hypothesis, first, correlation analysis was conducted, and it was found that the expression of miR-139-5p was negatively correlated with CTBP1 levels (Fig. 6A). Then a luciferase assay confirmed that miRNA-139-5p could inhibit the fluorescence activity of wild-type CTBP1 without affecting the fluorescence activity of mutant CTBP1, confirming the regulatory relationship between miRNA-139-5p and CTBP1 (Fig. 6B and C). Overexpression of miR-139-5p downregulated CTBP1 mRNA and protein expression levels, while silencing of miR-139-5p upregulated CTBP1 mRNA and protein expression levels (Fig. 6D–H). All these results indicated that miR-139-5p has a targeted regulatory relationship with CTBP1. In the drug resistance study, we co-transfected miR-139-5p mimics and CTBP1 overexpression plasmid into PC cells, which were subsequently treated with GEM. We found that CTBP1 could reverse the effects of miR-139-5p on PC cell invasion, migration, and GEM resistance (Fig. 7A–I). On the contrary, when miR-139-5p and CTBP1 were silenced at the same time, si-CTBP1 could significantly inhibit PC cell invasion and migration, stemness, and GEM resistance (Fig. 7J–O). Therefore, CTBP1 is an important molecule downstream of miR-139-5p, and miR-139-5p exerts tumorigenic effects by regulating CTBP1.

### SH3BP5-AS1 contributes to the effects of CTBP1 and increases tumor malignancy via the Wnt/β-catenin pathway

**Fig. 8:**
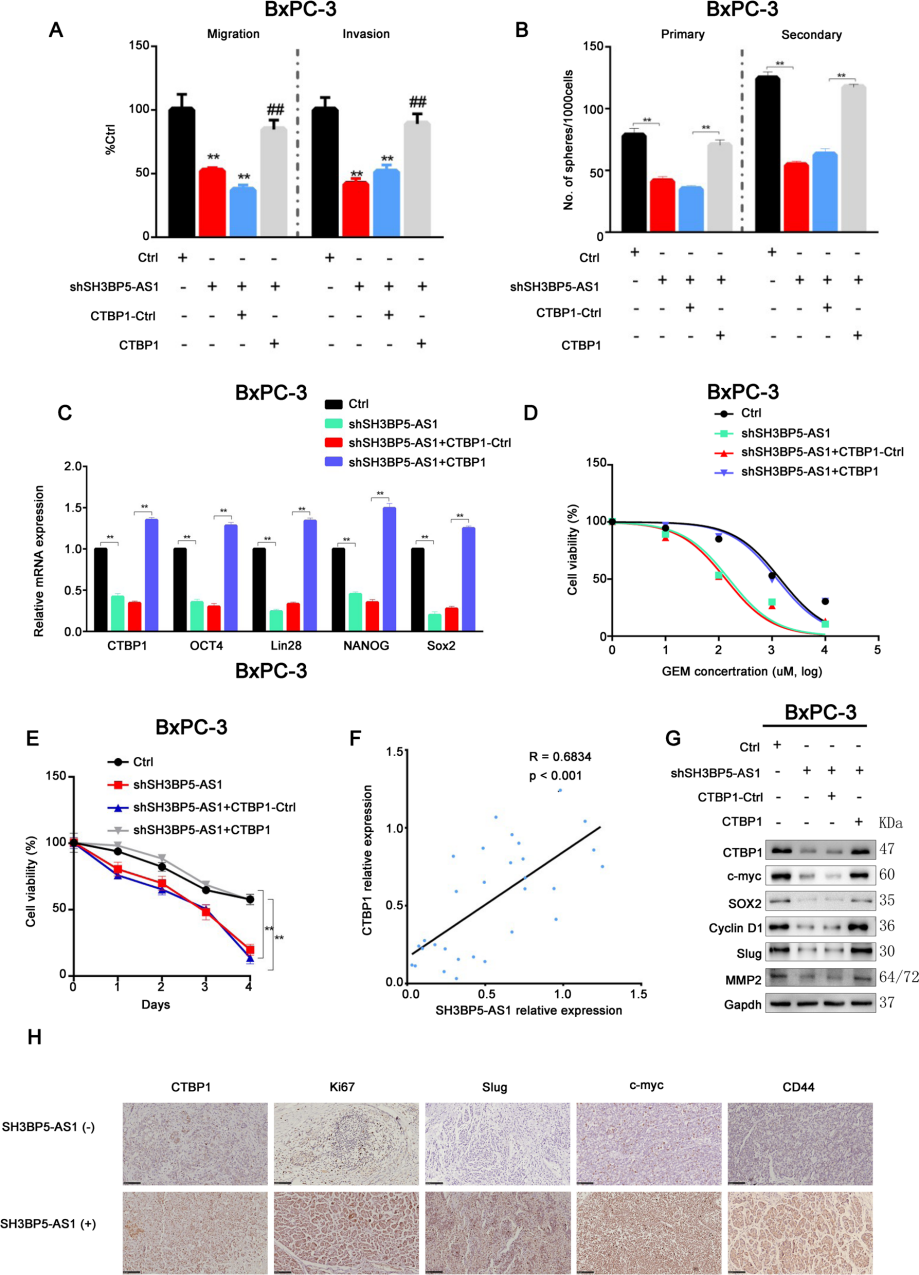
SH3BP5-AS1 contributes to CTBP1 expression and increases tumor malignancy via the Wnt/β-catenin pathway. **A**–**B** Transwell and Sphere formation assays were performed in BxPC-3 cells after co-transfection with SH3BP5-AS1-shRNA and/or CTBP1 overexpression plasmid. **C** Stem cell-like cell markers Lin28, OCT4, NANOG, and SOX-2 were detected by western blot following the indicated transfections in BxPC-3 cells. **D** BxPC-3 cells with indicated transfections were treated with GEM at different concentrations for 48 h. Cell viability was measured by MTT assay. **E** BxPC-3 cells with indicated transfections were treated with GEM (1 μM) for 48 h. Cell viability was measured by MTT assay at different timepoints. **F** Correlation analysis of CTBP1 and miRNA-139-5p. **G** The proteins of Wnt/β-catenin pathway were detected by western blot following the indicated transfections in BxPC-3 cells. **H** Proteins of the Wnt/β-catenin pathway were detected by IHC following the indicated transfections in BxPC-3 cells. Data are shown as the mean ± SD. *P < 0.05; **P < 0.01

SH3BP5-AS1 participates in the GEM resistance of PC cells through a ceRNA mechanism, as confirmed in the above described experiments. When we further analyzed the regulatory relationship between SH3BP5-AS1 and CTBP1, we found that SH3BP5-AS1 silencing reduced cell invasion, migration, and stemness and enhanced GEM sensitivity in PC cells, whereas CTBP1 could reverse the abovementioned effects of sh-SH3BP5-AS1 (Fig. 8A–C, Additional file 1: Fig. S6A, C and E). Conversely, silencing of CTBP1 could significantly inhibit the biological effects of SH3BP5-AS1 overexpression on PC cells (Additional file 1: Fig. S6B, D and H). Similarly, CTBP1 restored the effects of sh-SH3BP5-AS1 on GEM sensitivity in PC cells, as observed in the drug sensitivity assay (Fig. 8D and E, Additional file 1: Fig. S6F and G). Further correlation analysis revealed that the expression of SH3BP5-AS1 was positive correlated with CTBP1 levels (Fig. 8F).

According to our transcriptome sequencing results, the Wnt pathway might be an important signaling pathway for SH3BP5-AS1/miRNA-139-5p/CTBP1 to exert its effect. We further detected Wnt signaling pathway-related proteins through western blot and IHC and found that after silencing SH3BP5-AS1, the proteins of Wnt pathway were significantly down-regulated. When CTBP1 was overexpressed at the same time, the Wnt signaling pathway was activated (Fig. 8G and H, Additional file 1: Fig. S6K). Conversely, silencing of CTBP1 could significantly inhibit the effects of SH3BP5-AS1 on Wnt signaling pathway (Additional file 1: Fig. S6L). These results indicated that SH3BP5-AS1/miRNA-139-5p/CTBP1 might exert their biological functions via the Wnt signaling pathway.

## Discussion

**Fig. 9:**
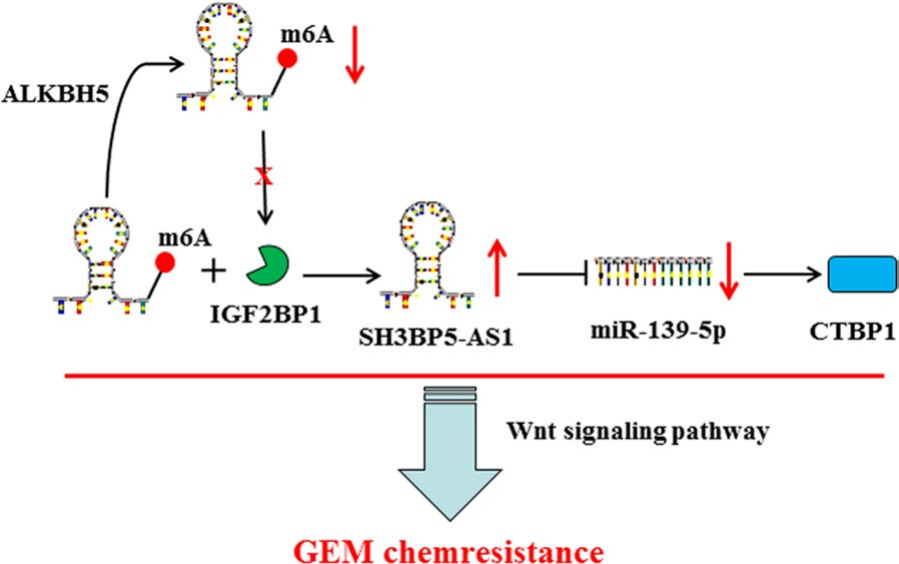
The schematic diagram of the carcinogenesis role of SH3BP5-AS1 in PC and its mechanism

Chemoresistance has always been a frequently encountered bottleneck in the treatment of PC. Many studies on the effects of lncRNA on tumor chemoresistance and the underlying mechanisms have been conducted, aiming to find a marker with good specificity and sensitivity [6, 24, 25]. In this study, a PDX model combined with high-throughput sequencing was used to screen the key lncRNAs in PC chemoresistance. SH3BP5-AS1 is closely related to PC drug resistance. Only two studies have reported that SH3BP5-AS1 might be related to inflammation and the prognosis of head and neck tumors [9, 26]; however, the role of SH3BP5-AS1 has not been studied in PC. It is important to fully elucidate the mechanism of SH3BP5-AS1 in the drug resistance of PC. In this study, we found that high SH3BP5-AS1 expression promoted the invasion, migration, and stemness of PC cells and increased the resistance of PC cells to GEM. Furthermore, this study also revealed that SH3BP5-AS1 activates the Wnt signaling pathway through a ceRNA mechanism, which affects carcinogenesis and chemoresistance in PC (Fig. 9).

More and more studies have shown that lncRNAs play important roles in the occurrence and development of malignant tumors including PC [6, 24, 27], This study found that the expression of SH3BP5-AS1 in PC (i) is significantly higher than that in normal pancreatic tissue and (ii) plays a role in promoting tumorigenesis. The results were also consistent with previous reports. For example, it has been reported that SH3BP5-AS1 (i) promotes the occurrence and development of head and neck cancer and (ii) is an independent risk factor for its prognosis [9]. In this study, correlation analysis of the clinicopathological characteristics and SH3BP5-AS1 expression suggested that high SH3BP5-AS1 expression is closely related to the TNM stage and poor prognosis of PC. This evidence proves the important role of SH3BP5-AS1 in PC. Moreover, we also found that SH3BP5-AS1 overexpression might be regulated by m6A modification. m6A modification has been confirmed to be involved in the regulation of gene stability. It participates in the occurrence of a variety of tumors by regulating RNA transcription, splicing, processing, translation, and degradation [28]. m6A modifications are established and removed by m6A methyltransferases (writers) or demethylases (erasers), respectively, and recognized by m6A-binding proteins (readers), which ultimately affects the expression levels of RNAs. There are many studies on the roles of m6A modification in PC. The results of this study suggested that the “eraser” ALKBH5 and the “reader” IGF2BP1 are involved in the regulation of SH3BP5-AS1 expression. ALKBH5 has been confirmed to be downregulated in PC, and low ALKBH5 expression promotes the occurrence and development of tumors [19, 29]. The clinical analyses of our center are consistent with previous reports. ALKBH5 expression was negatively correlated with SH3BP5-AS1 levels. Subsequent results confirmed that low ALKBH5 expression increased the level of SH3BP5-AS1 m6A modification, which is further recognized by the m6A recognition protein IGF2BP1, thereby promoting the expression of SH3BP5-AS1. This result also explains why SH3BP5-AS1 was highly expressed in PC. This is the first report of the regulatory mechanism of m6A modification of SH3BP5-AS1 in PC.

In this study, a new lncRNA, SH3BP5-AS1, affects GEM resistance in PC was discovered. A lack of SH3BP5-AS1 expression could enhance the sensitivity of PC cells to GEM. In vivo and in vitro experiments confirmed that SH3BP5-AS1 was involved in GEM resistance of PC. We hypothesized that it might exert different functions in different subcellular compartments. Our previous study confirmed that lncRNAs might exert their effects via both nuclear and cytoplasmic pathways [30]. Our experiments have proved that SH3BP5-AS1 is modified by m6A in the nucleus, leading to its high expression, and then part of SH3BP5-AS1 is transferred to the cytoplasm, further regulating miR-139-5p expression by the sponge effect. Our previous studies have confirmed that low expression of miR-139-5p promotes the occurrence and development of PC, and miR-139-5p has also been confirmed to participate in the drug resistance of tumors including non-small cell lung cancer, ovarian cancer, and colon cancer [24]. *CTBP1*, a downstream target gene of miR-139-5p, was screened by transcriptome sequencing in this study. CTBP1 has been reported to be involved in tumor chemoresistance [31]; its high expression led to the occurrence of EMT and promoted the invasion and migration of tumor cells [32]. The results of this study are consistent with the conclusions of the above studies. Our study revealed that high SH3BP5-AS1 expression further upregulated the expression of CTBP1 through downregulation of miR-139-5p, which ultimately led to GEM resistance in PC. However, whether SH3BP5-AS1 participates in other regulatory mechanisms in the nucleus has not been explored. Moreover, it remains to be elucidated whether SH3BP5-AS1 can directly regulate the expression of the downstream target gene *CTBP1* and how SH3BP5-AS1 exerts its regulatory effects.

To explore the mechanisms by which the SH3BP5-AS1/miR-139-5p/CTBP1 axis affects GEM resistance in PC cells, high-throughput sequencing and signal pathway enrichment analysis were conducted. We found that when SH3BP5-AS1 competitively bound miR-139-5p via ceRNA to regulate the downstream gene *CTBP1*, it further regulated the Wnt signaling pathway. The Wnt signaling pathway has been confirmed to be involved in the tumorigenesis and drug resistance of a variety of tumors, including PC [24]. Studies have found that activation of the Wnt signaling pathway promotes the resistance of PC to GEM and the aggressiveness of tumors.

CTBP1 is an essential molecule in the Wnt signaling pathway, which could affect cell proliferation and cell survival [33, 34]. The above results partially explain how abnormal SH3BP5-AS1 expression affects GEM resistance in PC cells and reveal its downstream regulatory mechanism. SH3BP5-AS1 may serve as a treatment target to enhance the sensitivity of PC to GEM.

This study revealed that SH3BP5-AS1 activated Wnt signaling pathway by sponging miR-139-5p, upregulating CTBP1 expression, and contributing to the sensitivity of PC cells to GEM. SH3BP5-AS1 is expected to a potential target for PC therapy.

## Conclusions

In summary, this study revealed that SH3BP5-AS1 activated Wnt signaling pathway by sponging miR-139-5p, upregulating CTBP1 expression, and contributing to the sensitivity of PC cells to GEM. SH3BP5-AS1 might be a potential target for PC therapy.

## Methods

### Clinical specimens

In total, 87 PC samples were collected from the First Affiliated Hospital of Fujian Medical University between 2008 and 2018. The clinicopathological characteristics including age, sex, alcohol history, smoking history, CEA, CA199, tumor size, tumor stage, lymph node invasion, and metastasis were recorded. We have obtained informed consent from all patients and this study was approved by the Ethics Committee of the First Affiliated Hospital of Fujian Medical University (Fujian, China).

### Establishment of patient derived xenograft (PDX) model

The fresh tumor fragments were subcutaneously transplanted into the left flank of anaesthetized NOD scid gamma mice. Mice were observed for maximum 120 d and keep feeding under sterile and controlled conditions (22 °C, 50% relative humidity, 12 h light–dark cycle, autoclaved food and bedding, acidified drinking water). Subcutaneous tumor growth was measured in 2 dimensions using a caliper. Tumor volumes (TV) were determined by the formula: TV = (length x width^2^)/2. Tumors were routinely passaged when the TV approximately grow in 1 cm^3^. Xenograft material was snap frozen and stored at -80 °C or processed to formalin fixed, paraffin embedded (FFPE) blocks.

### Cell culture

The human PC cell lines AsPC-1, CFPAC-1, Capan-1, SW1990, PANC-1, SW1990, BxPC-3, and MIA-PaCa-2 and the non-cancerous HPDE6C7 cell line were purchased from the American Type Culture Collection (ATCC; Manassas, VA, USA). Cells were cultured in Dulbecco’s modified Eagle’s medium (DMEM) supplemented with 10% fetal bovine serum (FBS) and 1% antibiotic/antifungal solution (Biowest, Nuaillé, France) in 5% CO_2_ at 37 °C in a humidified atmosphere.

### Immunohistochemistry

Tissues were fixed with 10% formalin for about 48 h and paraffin-embedded for immunohistochemistry (IHC) assays. The protocol was previously described [10]. All antibodies and related reagents are shown in Additional file 1: Table S1.

### Western blot

Proteins were extracted from homogenized tissues or cultured cells and quantified by using the BCA method, sample buffer was added, samples were heated, and proteins were separated by SDS-PAGE and transferred to a polyvinylidene fluoride membrane (Bio-Rad Laboratories, Hercules, CA, USA). The membrane was blocked in 5% skim milk for about 1 h and incubated with primary antibody (Additional file 1: Table S1) at 4 °C overnight. Then the membrane was washed and incubated with secondary antibody for 2 h, and after washing, protein bands were visualized using enhanced chemiluminescence (ECL) substrate and photographed. Relative expression levels of proteins were quantified by ImageJ software version 1.8.0 (National Institutes of Health, Bethesda, MD, USA).

### Quantitative reverse-transcriptase PCR

Total RNA was isolated using TRIzol reagent (Invitrogen, Carlsbad, CA, USA) following the manufacturer’s instructions, and cDNA was synthesized with random primers (Additional file 1: Table S2) using a reverse transcription kit (TOYOBO, Japan) following the manufacturer’s instructions. qRT-PCR was performed using Fast Start Universal SYBR Green Master Mix (Roche Diagnostics GmbH, Mannheim, Germany). The RNA level was quantifified by using the 2^−ΔΔCt^ method. All the primers that used in this study was showed in Additional file 1: Table S2.

### Colony formation assay

Cells were cultured in 6-well plates (500 cells/well) for 14 days. Then, the colonies were fixed by methanol/acetic acid (3:1, v/v) and stained with 0.5% crystal violet (Sigma-Aldrich, St. Louis, MO, USA). The colonies were counted using a microscope (Olympus IX81).

### Sphere formation assay

The Cells were inoculated in ultralow attachment six well plates (Corning) at a density of 1000 cells/well, and allow to culture for 1 week (Primary spheres) in DMEM/F12 medium (Invitrogen, Shanghai, China) supplemented with 20 ng/ml EGF (Sigma, Shanghai, China), B27 (1:50, GIBCO, Shanghai, China), and 20 ng/ml bFGF (Sigma, Shanghai, China). Subsequently, the primary spheres were collected, separated with trypsin and then seeded again to form secondary spheres. Finally, the number of the spheres was counted by using a microscope.

### RNA immunoprecipitation assay

The RNA immunoprecipitation (RIP) assay was performed using a Magna RIP™ RNA-Binding Protein Immunoprecipitation Kit (Millipore, USA). Briefly, cell lysates were immunoprecipitated with beads conjugated with anti-AGO2 or anti-IgG at 4 °C for 6 h. Then, the immunoprecipitates were treated with 0.1% SDS/Proteinase K (0.5 mg/mL, 30 min at 55 °C), and immunoprecipitated RNAs were detected by qRT-PCR.

### Methylated RNA immunoprecipitation qPCR

An miRNeasy kit (#217004, QIAGEN) was used for isolating the total RNA fraction. The GenElute mRNA Miniprep kit (MRN10, Sigma-Aldrich) was used for extracting mRNA from the total RNA fraction. Then, a Magna MeRIP m6A kit (#17–10,499, Millipore) was used according to the manufacturer’s instructions, and m6A enrichment was analyzed by qPCR.

### RNA pull-down assay

A Pierce Magnetic RNA–Protein Pull-Down Kit (Thermo Fisher Scientific, 20,164) was used for the RNA pull-down assay. Briefly, cell extracts were treated with RNase-free DNase I and incubated with biotinylated SH3BP5-AS1 in the presence of streptavidin magnetic beads, which can capture the proteins/miRNAs potentially interacting with SH3BP5-AS1. A Pierce™ RNA 3' End Desthiobiotinylation Kit (Thermo, 20,163) was used for SH3BP5-AS1 biotinylation labeling. Proteins and RNAs in the captured protein–RNA complex were analyzed using western blot and RT-qPCR, respectively.

### Drug sensitivity assay

To assess the chemosensitivity of PC cells to GEM, cells were treated with different concentrations of GEM (0.1, 1, 10, and 100 μM) for 72 h in 96-well plates as previously described [7]. An MTT kit was used to detect the number of viable cells, and the half maximal inhibitory concentration (IC_50_) was calculated to assess drug sensitivity.

### Overexpression plasmid and siRNA transfection

The overexpression plasmid and small interfering RNA (siRNA) were purchased from GenePharma (Shanghai, China). Cells at a concentration of 5 × 10^5^ cells/ml per well were cultured in 6-well plates for 2 days. PC cells were transfected with overexpression plasmid or negative controls using Lipofectamine 3000 (Invitrogen) at a final concentration of 50 nM. Cells were transfected with siRNAs or si-NCs using Lipofectamine 3000 (Invitrogen) at a final concentration of 100 nM.

### Cell migration and invasion assay

The Transwell assay was used to analyze cell migration and invasion. Chambers coated with Matrigel (Corning Labware Products Inc., Corning, NY, USA) were used for invasion assays, and uncoated chambers with Matrigel were used for cell migration assays. First, 500 μl basal medium (without serum) was added into the chambers. After incubation for 2 h, the chambers were placed in a 24-well plate containing cell culture medium with 10% FBS. Then, 100 μl cell suspension (2 × 10^5^ cells/ml) was added into the chamber. After 24 h, the chambers were fixed with paraformaldehyde and then stained with crystal violet to allow visualization. Cells were photographed under an Olympus IX73 microscope (Olympus).

### In vivo assay

Six-week-old male BALB/c nude mice were purchased from the Shanghai Laboratory Animal Center for in vivo experiments. Cells stably transfected with the sh-SH3BP5-AS1 lentivirus or empty vectors were injected subcutaneously or into the tail vein of the selected mice. After 4 weeks, the tumors were harvested and their weight and volume were measured. The experimental protocols were approved by the Fujian Medical University Ethics Review Committee.

### Statistical analysis

GraphPad software v.6 and SPSS software version 21.0 (IBM Corp., Armonk, NY, USA) were used for statistical analysis. Measured data are reported as the mean ± standard deviation (SD). Student’s *t*-test or one-way ANOVA was used to evaluate means between groups. Pearson correlation analysis was performed to determine correlations. Survival was analyzed by Kaplan–Meier curves. The correlations between expression levels and clinicopathological features were analyzed using either the χ^2^ test or Fisher’s exact test. Data are reported from at least three independent experiments. *P* < 0.05 was considered to be statistically significant.

## Supplementary Information

**Additional file 1**. Supplementary figures and materials.

## Data Availability

All the data obtained in current study were available from the corresponding authors on reasonable request.

## Abbreviations

m6A: N6-methyladenosine
lncRNA: Long non-coding RNA
PC: Pancreatic cancer
PDX: Patient-derived xenograft
shRNA: Short hairpin RNA
DMEM: Dulbecco’s modified eagle’s medium
CTBP1: C-terminal binding protein 1
FBS: Fetal bovine serum
IHC: Immunohistochemistry
RIP: Immunoprecipitation
MeRIP: Methylated RNA immunoprecipitation qPCR
