# Supplementary material for: N6-methyladenosine-mediated SH3BP5-AS1 upregulation promotes GEM chemoresistance in pancreatic cancer by activating the Wnt signaling pathway
Source: Biol Direct. 2022 Nov 17;17:33. doi: 10.1186/s13062-022-00347-5 (PMC9673340; doi:10.1186/s13062-022-00347-5)
Supplement: Supplementary file 1 — Additional file 1. Supplementary figures and materials. [file 13062_2022_347_MOESM1_ESM.zip › Supplementary files/Supplementary table 2 .doc]

Supplementary table 2. The information of primers used in this study

| Gene name |  | Sequece (5’—3’) |
| --- | --- | --- |
| GAPDH | Forward | GGAGTCCACTGGCGTCTTCA |
|  | Reverse | GTCATGAGTCCTTCCACGATACC |
| SH3BP5-AS1 | Forward | CCGCTCGTGGATCTCATCTG |
|  | Reverse | GCATCTTAAGGCTAGCAGGGT |
| OCT4 | Forward | CAAAGCAGAAACCCTCGTGC |
|  | Reverse | AACCACACTCGGACCACATC |
| Lin28A | Forward | CAACCAGCAGTTTGCAGGTGG |
|  | Reverse | GCGGTCATGGACAGGAAGCC |
| NANOG | Forward | GATGCCTCACACGGAGACTG |
|  | Reverse | TTGACCGGGACCTTGTCTTC |
| ALKBH5 | Forward | TCGTGTCCGTGTCCTTCTTT |
|  | Reverse | GATGTCCTGAGGCCGTATGC |
| IGF2BP1 | Forward | AAGACCTTACCCTTTACAACCC |
|  | Reverse | GCAGCCACATCATTCTCATAG |
| CTBP1 | Forward | CGACCCTTACTTGTCGGATGG |
|  | Reverse | TTGACGGTGAAGTCGTTGATG |
| has-miR-139-5p | Forward | GTCGTATCCAGTGCAGGGTCCGAGG |
|  | Reverse | TATTCGCACTGGATACGACCTGGAG |
| U6 | Forward | CTCGCTTCGGCAGCACA |
|  | Reverse | AACGCTTCACGAATTTGCGT |
