# Supplementary material for: N6-methyladenosine-mediated SH3BP5-AS1 upregulation promotes GEM chemoresistance in pancreatic cancer by activating the Wnt signaling pathway
Source: Biol Direct. 2022 Nov 17;17:33. doi: 10.1186/s13062-022-00347-5 (PMC9673340; doi:10.1186/s13062-022-00347-5)
Supplement: Supplementary file 1 — Additional file 1. Supplementary figures and materials. [file 13062_2022_347_MOESM1_ESM.zip › Supplementary files/Supplementary table 3.docx]

Supplementary table 3. The information of short interfering RNA (siRNA) used in this study

| Short interfering RNA | | Sequence information | Complany |
| --- | --- | --- | --- |
| shSH3BP5-AS1 | shSH3BP5-AS1#1 | 5′-GCTCAAACCAGTCAGCTAACT-3′ | Genechem  (Shanghai, China) |
|  | shSH3BP5-AS1#2 | 5'- GCAGGATCCCATGGATATTAA-3' |  |
| shALKBH5 | shCtrl | 5′- TTCTCCGAACGTGTCACGT -3′ | Gene Parma  (Shanghai, China) |
|  | shALKBH5#1 | 5'- GACGTCCCGGGACAACTATAA -3' |  |
|  | shALKBH5#2 | 5'- GACTGTGCTCAGTGGATATGC-3' |  |
| siIGF2BP1 | siIGF2BP1 #1 | 5’- AUG​AAA​CAU​AAC​UUU​CUU​GUU-3′ | Gene Parma  (Shanghai, China) |
|  | siIGF2BP1 #2 | 5′- UUA​AUC​UAC​AGA​UAC​UGA​CAG-3′ |  |
|  | siIGF2BP1 #3 | 5′- AGC​AUU​UUU​UUU​AAG​UCA​CUC-3′ |  |
| siCTBP1 | siCTBP1#1 | 5' -GGAUAGAGACCACGCCAGU- 3' | Gene Parma  (Shanghai, China) |
|  | siCTBP1#2 | 5' -GAGCAGGCAUCCAUCGAGA- 3' |  |
|  | siCTBP1#3 | 5' -UGAAGAACUGUGUCAACAA- 3' |  |
