# Supplementary figures and images for: N6-methyladenosine-mediated SH3BP5-AS1 upregulation promotes GEM chemoresistance in pancreatic cancer by activating the Wnt signaling pathway
Source: Biol Direct. 2022 Nov 17;17:33. doi: 10.1186/s13062-022-00347-5 (PMC9673340; doi:10.1186/s13062-022-00347-5)

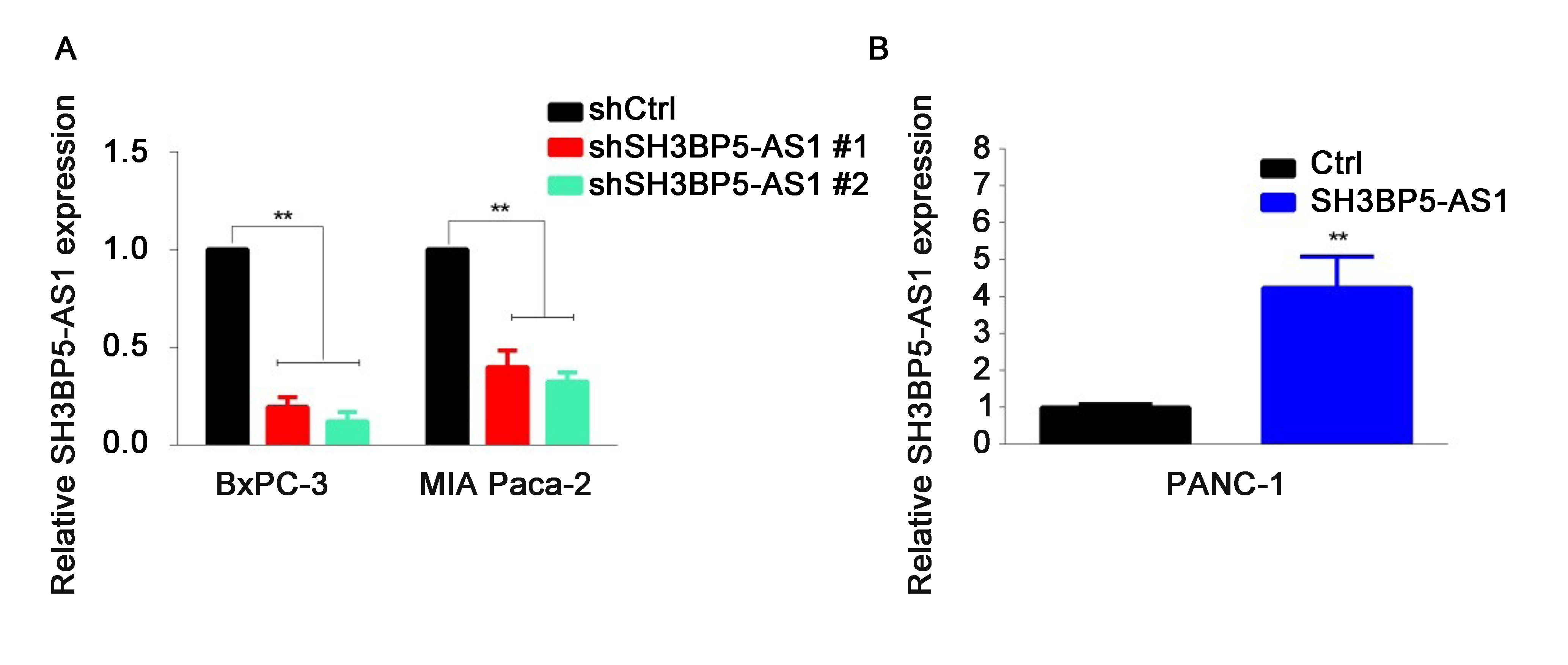

Supplement: Supplementary file 1 — Additional file 1. Supplementary figures and materials. [file 13062_2022_347_MOESM1_ESM.zip › Supplementary files/Sfig 1.tif]

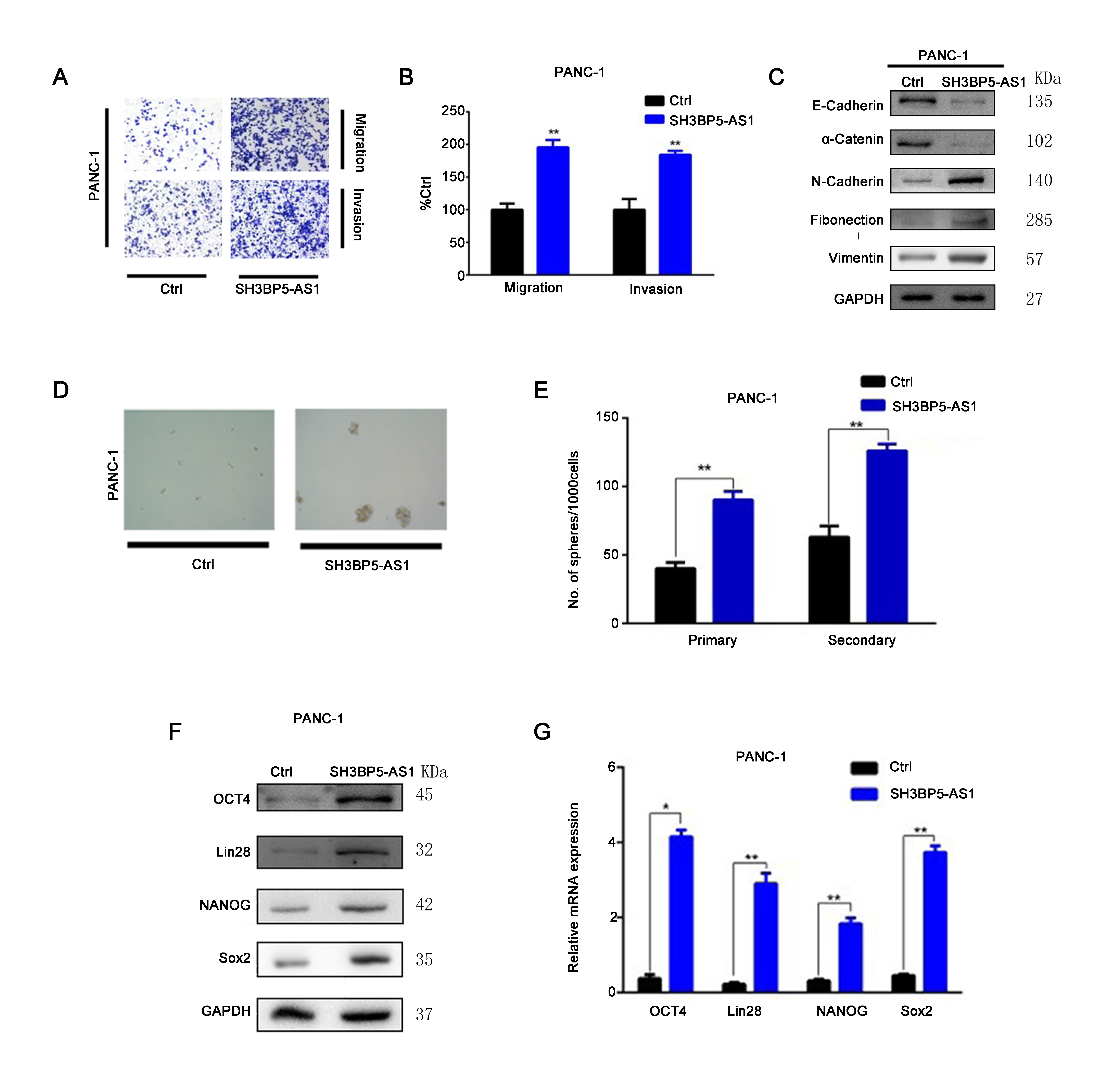

Supplement: Supplementary file 1 — Additional file 1. Supplementary figures and materials. [file 13062_2022_347_MOESM1_ESM.zip › Supplementary files/Sfig 2.tif]

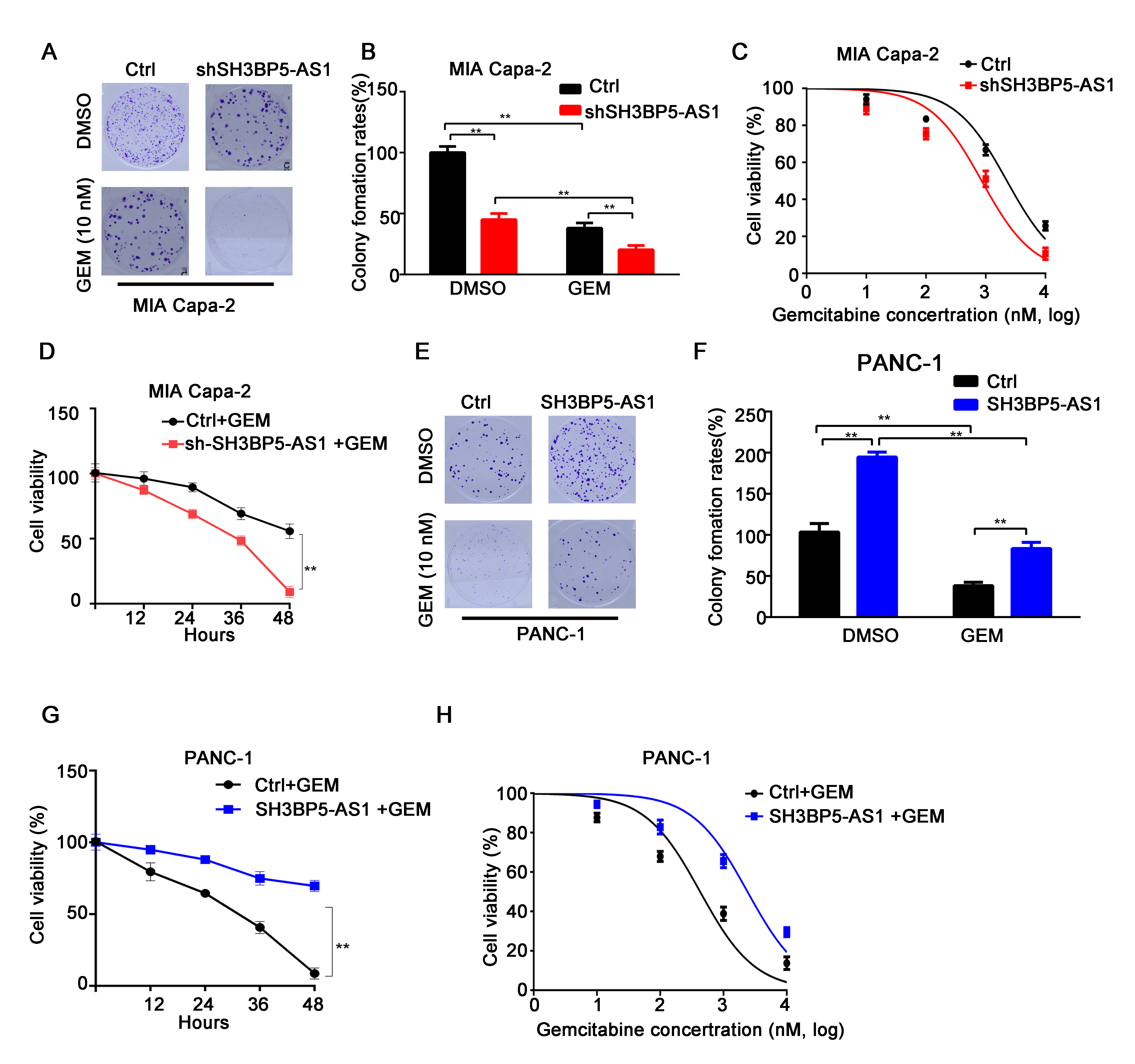

Supplement: Supplementary file 1 — Additional file 1. Supplementary figures and materials. [file 13062_2022_347_MOESM1_ESM.zip › Supplementary files/Sfig 3.tif]

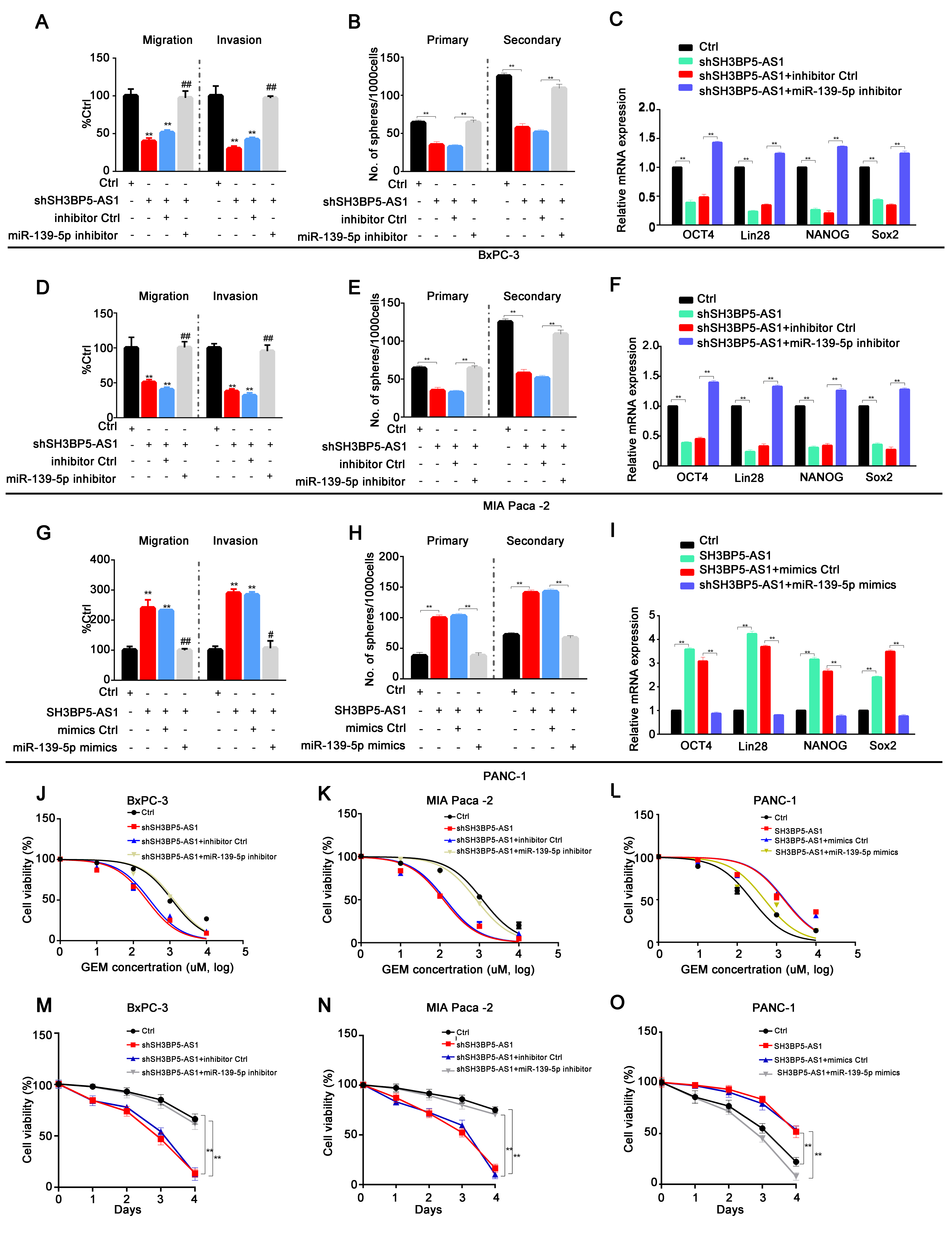

Supplement: Supplementary file 1 — Additional file 1. Supplementary figures and materials. [file 13062_2022_347_MOESM1_ESM.zip › Supplementary files/Sfig 4.tif]

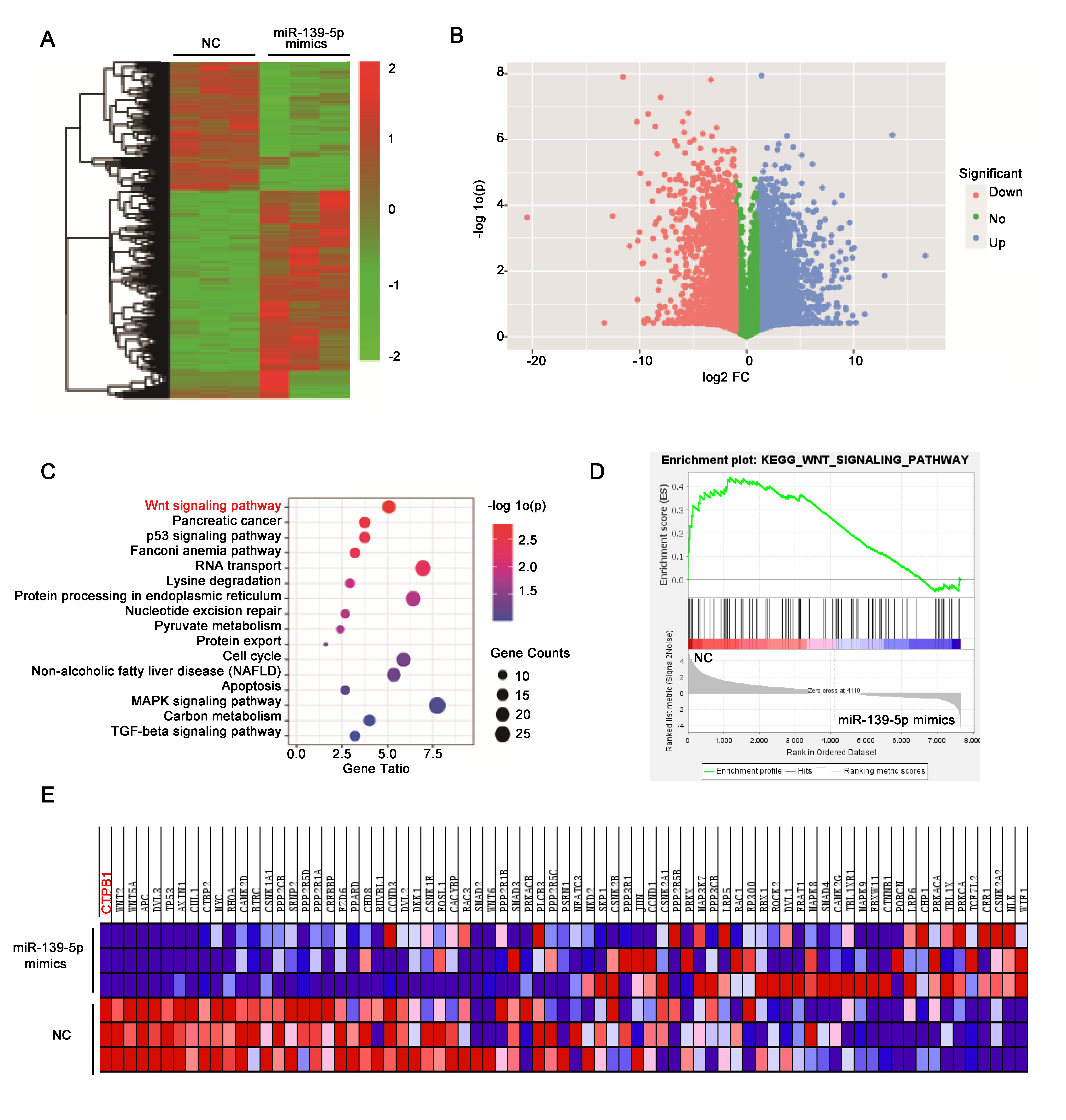

Supplement: Supplementary file 1 — Additional file 1. Supplementary figures and materials. [file 13062_2022_347_MOESM1_ESM.zip › Supplementary files/Sfig 5 .tif]

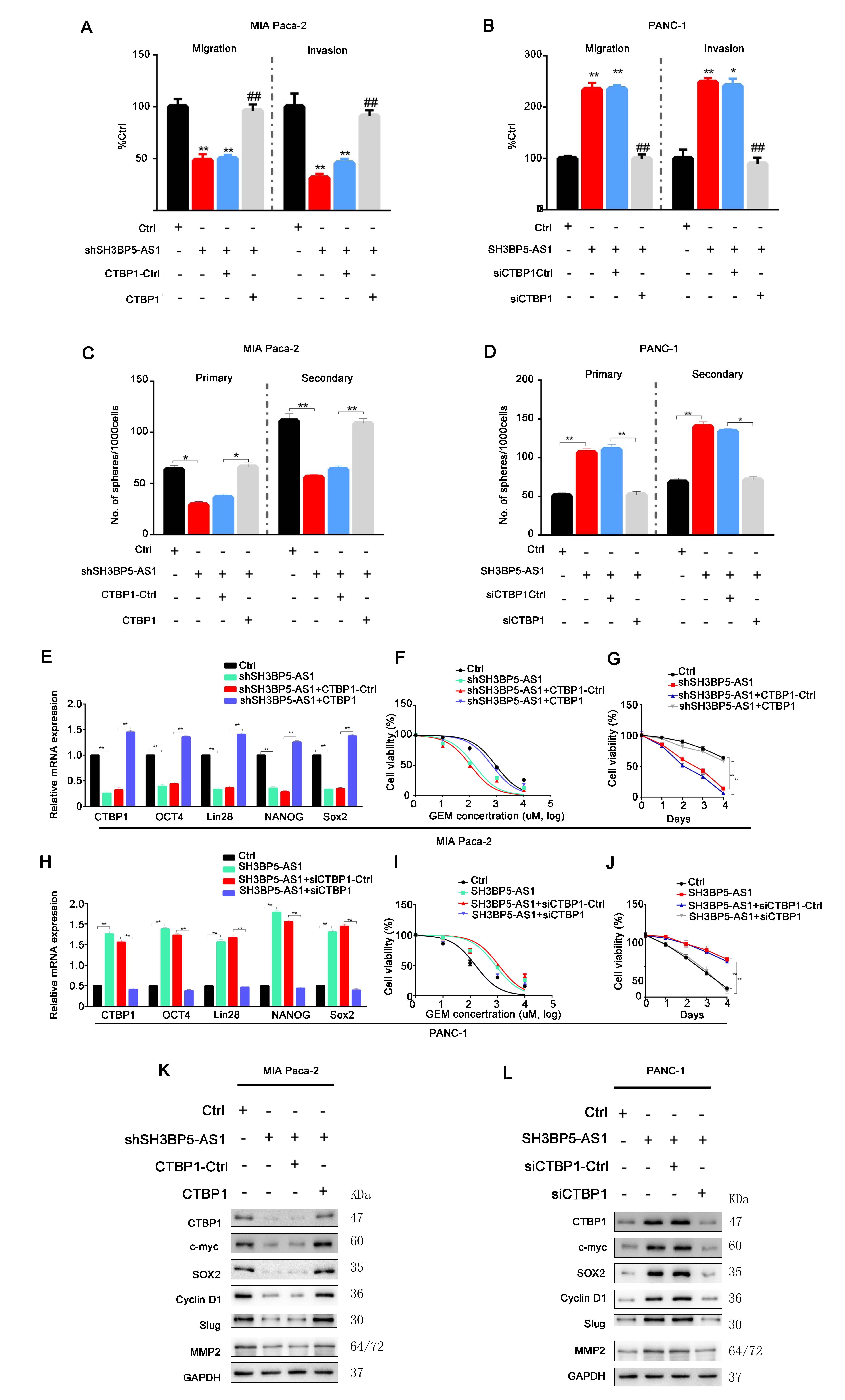

Supplement: Supplementary file 1 — Additional file 1. Supplementary figures and materials. [file 13062_2022_347_MOESM1_ESM.zip › Supplementary files/Sfig 6.tif]
