# Supplementary material for: N6-methyladenosine-mediated SH3BP5-AS1 upregulation promotes GEM chemoresistance in pancreatic cancer by activating the Wnt signaling pathway
Source: Biol Direct. 2022 Nov 17;17:33. doi: 10.1186/s13062-022-00347-5 (PMC9673340; doi:10.1186/s13062-022-00347-5)
Supplement: Supplementary file 1 — Additional file 1. Supplementary figures and materials. [file 13062_2022_347_MOESM1_ESM.zip › Supplementary files/Supplementary figure legends.doc]

**Supplementary figure legends**

**Figure S1. SH3BP5-AS1 expression after transfection with shSH3BP5-AS1 or SH3BP5-AS1 overexpression plasmid.** (A) SH3BP5-AS1 expression in BxPC-3 and MIA Paca-2 cells transfected with shCtr or shSH3BP5-AS1. (B) SH3BP5-AS1 expression in PANC-1 cells transfected with the control vector or SH3BP5-AS1. Data are shown as the mean ± SD. ^**^*P* < 0.01.

**Figure S2. SH3BP5-AS1 overexpression promoted the invasion, migration, and stemness of pancreatic cancer cells in vitro.**

(A, B) Transwell assay displaying the migration and invasion of PANC-1 cells transfected with empty vector or SH3BP5-AS1. (C) EMT-related markers were detected by western blot. (D, E) Sphere formation analysis. (F, G) Stem cell-like cell markers OCT4, Lin28, NANOG, and SOX-2 were detected by western blot. Data are shown as the mean ± SD of three replicates. **P* < 0.05; ***P* < 0.01.

**Figure S3. Abnormal expression of SH3BP5-AS1 affected the sensitivity of pancreatic cancer cell to gemcitabine.**

(A, B) Colony formation in MIA Paca-2 cells transfected with shCtr or shSH3BP5-AS1 and treated with DMSO or GEM. (C) MIA Paca-2 cells with stable SH3BP5-AS1 silencing were treated with GEM at different concentrations for 48 h. Cell viability was measured by MTT assay. (D) MIA Paca-2 cells with stable SH3BP5-AS1 silencing were treated with GEM (1 μM) for 48 h. Cell viability was measured by MTT assay at different timepoints. (E, F) Colony formation in PANC-1 cells transfected with empty vector or SH3BP5-AS1 and treated with DMSO or GEM. (G) PANC-1 cells with stable SH3BP5-AS1 silencing were treated with GEM at different concentrations for 48 h. Cell viability was measured by MTT assay. (H) PANC-1 cells with stable SH3BP5-AS1 silencing were treated with GEM (1 μM) for 48 h. Cell viability was measured by MTT assay at different timepoints.

**Figure S4. SH3BP5-AS1 promotes pancreatic cancer cell resistance to gemcitabine by targeting miR-139-5p.**

(A, B) Transwell and Sphere formation assays were performed in BxPC-3 cells after co-transfection with shSH3BP5-AS1 and shRNA targeting miR-139-5p. (C) Stem cell-like cell markers Lin28, OCT4, NANOG, and SOX-2 were detected by western blot following the indicated transfections in BxPC-3 cells. (D, E) Transwell and Sphere formation assays were performed in MIA Paca-2 cells following the indicated transfections. (F) Stem cell-like cell markers Lin28, OCT4, NANOG, and SOX-2 were detected by western blot following the indicated transfections in MIA Paca-2 cells. (G, H) Transwell and Sphere formation assays were performed in PANC-1 cells after co-transfection with SH3BP5-AS1 and/or miR-139-5p mimics. (I) Stem cell-like cell markers Lin28, OCT4, NANOG, and SOX-2 were detected by western blot following the indicated transfections in PANC-1 cells. (J–L) Pancreatic cancer cells with indicated transfections were treated with GEM at different concentrations for 48 h. Cell viability was measured by MTT assay. (M–O) Pancreatic cancer cells with indicated transfections were treated with GEM (1 μM) for 48 h. Cell viability was measured by MTT assay at different timepoints. Data are shown as the mean ± SD. **P* < 0.05; ***P* < 0.01.

**Figure S5. Differentially expressed genes were screened by transcriptional sequencing following transfection with NC or miR-139-5p mimics.** (A) Heatmap. (B) Volcano plot. (C) KEGG analysis showed miR-139-5p might exert its effects via the Wnt signaling pathway. (D) Gene set enrichment analysis (GSEA) of the differentially expressed genes. (E) Differentially expressed genes enriched in the Wnt signaling pathway.

**Figure S6. Abnormal expression of SH3BP5-AS1 interfered with CTBP1 expression, affecting tumor malignancy via the Wnt/β-catenin pathway.**

(A, B) The Transwell assay was performed in MIA Paca-2 and PANC-1 cells after co-transfection with SH3BP5-AS1-shRNA/SH3BP5-AS1 and/or CTBP1 overexpression plasmid/CTBP1-siRNA. (C, D) A Sphere formation assay was performed in MIA Paca-2 and PANC-1 cells after the indicated transfections. (E) Stem cell-like cell markers Lin28, OCT4, NANOG, and SOX-2 were detected by western blot following the indicated transfections in MIA Paca-2 cells. (F) MIA Paca-2 cells with indicated transfections were treated with GEM at different concentrations for 48 h. Cell viability was measured by MTT assay. (G) MIA Paca-2 cells with indicated transfections were treated with GEM (1 μM) for 48 h. Cell viability was measured by MTT assay at different timepoints. (H) Stem cell-like cell markers Lin28, OCT4, NANOG, and SOX-2 were detected by western blot following the indicated transfections in PANC-1 cells. (I) PANC-1 cells with indicated transfections were treated with GEM at different concentrations for 48 h. Cell viability was measured by MTT assay. (J) PANC-1 cells with indicated transfections were treated with GEM (1 μM) for 48 h. Cell viability was measured by MTT assay at different timepoints. (K, L) Proteins of the Wnt/β-catenin pathway were detected by western blot following the indicated transfections in MIA Paca-2 and PANC-1 cells. Data are shown as the mean ± SD. **P* < 0.05; ***P* < 0.01.
