# Supplementary material for: N6-methyladenosine-mediated SH3BP5-AS1 upregulation promotes GEM chemoresistance in pancreatic cancer by activating the Wnt signaling pathway
Source: Biol Direct. 2022 Nov 17;17:33. doi: 10.1186/s13062-022-00347-5 (PMC9673340; doi:10.1186/s13062-022-00347-5)
Supplement: Supplementary file 1 — Additional file 1. Supplementary figures and materials. [file 13062_2022_347_MOESM1_ESM.zip › Supplementary files/Supplementary table 1.doc]

**Supplementary** table 1. The antibodies used in this study.

| Antibody | Company | Lot. No. |
| --- | --- | --- |
| GAPDH | Cell signaling technology | #5174 |
| E-cadherin | Cell signaling technology | #3195 |
| α-Catenin | Becton Dickinson |  |
| N-cadherin | Cell signaling technology | #13116 |
| Fibronectin | Abcam | ab2413 |
| Vimentin | Cell signaling technology | #5741 |
| OCT4 | Cell signaling technology | #2750 |
| Lin28 | Affinity Biosciences | #AF4072 |
| NANOG | Cell signaling technology | #4903 |
| SOX-2 | Cell signaling technology | #2748 |
| Ki67 | Abcam | ab15580 |
| CTBP1 | Cell signaling technology | #8684 |
| c-MYC | Cell signaling technology | #18583 |
| CyclinD1 | Cell signaling technology | #55506 |
| Slug | Abcam | ab27568 |
| MMP2 | Cell signaling technology | #40994 |
| CD44 | Affinity Biosciences | #DF6392 |
